# Integrative analysis of DNA, macroscopic remains and stable isotopes of dog coprolites to reconstruct community diet

**DOI:** 10.1038/s41598-021-82362-6

**Published:** 2021-02-04

**Authors:** Kelsey E. Witt, Karthik Yarlagadda, Julie M. Allen, Alyssa C. Bader, Mary L. Simon, Steven R. Kuehn, Kelly S. Swanson, Tzu-Wen L. Cross, Kristin M. Hedman, Stanley H. Ambrose, Ripan S. Malhi

**Affiliations:** 1 Program in Ecology, Evolution and Conservation Biology, University of Illinois at Urbana-Champaign, Urbana-Champaign, IL USA; 2 Ecology and Evolutionary Biology and Center for Computational and Molecular Biology, Brown University, Providence, RI USA; 3 Department of Anthropology, University of Illinois at Urbana-Champaign, Urbana-Champaign, IL USA; 4 Biology Department, University of Nevada Reno, Reno, NV USA; 5 Sealaska Heritage Institute, Juneau, AK USA; 6 Illinois State Archaeological Survey, Prairie Research Institute, University of Illinois at Urbana-Champaign, Urbana, IL USA; 7 Department of Animal Sciences, University of Illinois at Urbana-Champaign, Urbana, IL USA; 8 Division of Nutritional Sciences, University of Illinois at Urbana-Champaign, Urbana, IL USA; 9 Department of Veterinary Clinical Medicine, University of Illinois at Urbana-Champaign, Urbana, IL USA; 10 Carl R. Woese Institute for Genomic Biology, University of Illinois at Urbana-Champaign, Urbana, IL USA

**Keywords:** Genomic analysis, Bioinformatics

## Abstract

Paleofeces or coprolites are often used to reconstruct diet at archaeological sites, usually using macroscopic analyses or targeted DNA amplification and sequencing. Here we present an integrative analysis of dog coprolites, combining macroscopic analyses, stable isotope measurements, and DNA shotgun sequencing to examine diet and health status. Dog coprolites used in this study were recovered from the Janey B. Goode and East Saint Louis archaeological sites, both of which are located in the American Bottom, an extensive Mississippi River floodplain in Southwestern Illinois. Based on the context of recovery, coprolites are assigned to the Late Woodland and Terminal Late Woodland periods (ca. 600–1050 AD). Given the scarcity of human remains from this time period, these dog coprolites can be useful as a proxy for understanding human diet during the Late Woodland period. We find that the Late Woodland dogs consumed a variety of fish as well as bird and plant taxa, possibly including maize, and also harbored intestinal parasites and pathogenic bacteria. By sequencing the fecal microbiome of the coprolites, we find some similarities to modern dog microbiomes, as well as specific taxa that can be used to discriminate between modern and ancient microbiomes, excluding soil contaminants. As dogs are often used as a surrogate to assess human diet, humans living with these dogs likely had a similar diet and were affected by similar parasites. These analyses, when integrated, show a more comprehensive view of ancient dog and human diet and health in the region during the initial expansion of maize agriculture than any individual method could alone.

## Introduction

Ancient fecal material, known as coprolites and paleofeces, has been recovered from archaeological contexts worldwide and contain a wealth of information about the organism that produced them and the environment they lived in^1^. For example, pollen, seeds, animal bones, fish scales, and other plant and animal matter can be recovered from coprolites to reveal the types of organisms consumed^2,3^. Parasites and their eggs in coprolites can provide insight into the health of the animal that hosted them^4–6^. Coprolites can contain organic matter that can be used in radiocarbon dating to determine when an archaeological site was occupied^7–10^. The stable carbon and nitrogen isotope composition (δ^13^C and δ^15^N) of feces and coprolites can also be used to reconstruct diet isotopic composition^11–15^, and ancient bat guano and soil samples that likely contain fecal matter have been used to identify changes in climate, population demographics, and diet^16–19^.

Ancient DNA extraction and sequencing techniques can also be applied to coprolites, yielding even more information about the diets of the individuals who deposited them. DNA sequencing of coprolites has clarified the diet of multiple species, including ground sloths^7,20,21^, cave hyena^22^, moa^9,23–25^, and domestic dogs^26^. Plant and animal remains recovered from coprolites are fragmentary and often difficult to identify taxonomically; DNA analysis can help with more specific identifications, and can complement identifications made macroscopically or using pollen or phytoliths. Endogenous DNA can also be recovered from coprolites to identify the species that produced it^7,9,21,23,27^ and details of their population structure^28^. Additionally, coprolites contain a record of the fecal microbiome—the assemblage of bacteria and other microbes that live in the feces. If coprolites are sufficiently well-preserved, their microbiomes can show some similarities to modern microbiomes^29,30^, and can be used to identify dietary or cultural differences among ancient human populations^31,32^.

The majority of ancient DNA studies of coprolites have targeted specific regions of the genome for analyses^7,9,10,20,23,24,30^. Metabarcoding methods are often used to sequence a single region of a genome that is both short and highly variable between taxa. Different regions of the genome are used to identify different taxa (for a brief review of metabarcoding, see Creer et al.^33^). However, some taxa cannot be identified on the basis of a single region alone, and numerous DNA fragments from elsewhere in the genome are discarded when these methods are employed. Shotgun sequencing or metagenomics (which sequences all DNA in the sample) have been used in a small number of coprolite studies, but they either focused on specific taxa^29,34^ or only analyzed mitochondrial DNA reads following sequencing^22^.

Coprolites can be analyzed using multiple methods, as outlined above, and combining the results of these methods can provide a comprehensive view of an organism’s diet and health. Identifying fragmentary plant and animal remains from coprolites can complement the findings generated by ancient DNA analyses, and each method often provides complimentary insights into an organism’s diet^9,21,26^. Coprolites represent a record of only a few meals. While they can provide a lot of information of an organism’s diet and health spanning a few days, they cannot give a full picture of diet or environment across the life of that organism. Stable isotope analyses can be used to infer the protein sources and types of plants consumed, and provide a complementary, broader view of an organism’s diet. Recent studies of coprolites, bone and dental calculus using a combination of archaeological, isotopic and genetic methods revealed a wealth of information about ancient human populations in Italy^35^, Argentina^36^, Puerto Rico^37^, and eastern North America^38^.

**Figure 1:**
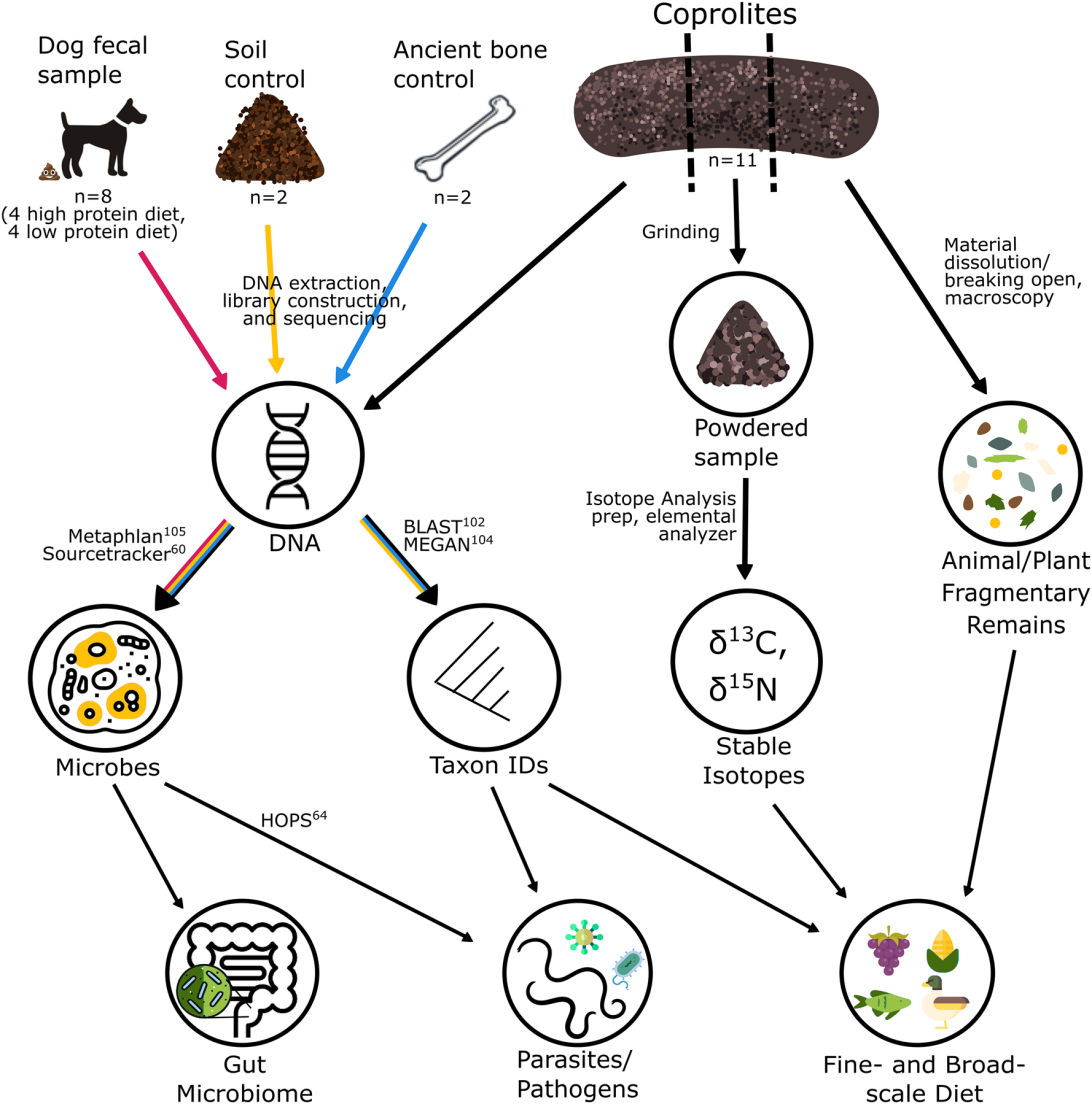
A graphic illustration of our pipeline for inferring diet and microbiome from modern dog feces and archaeological coprolites. The sample size for the coprolites and the controls are indicated underneath each sample type. Controls have color-coded arrows to indicate which controls were used for which analysis method. Arrows are labeled with the methods used to process the sample or analyze the results, and the reference is provided for the software mentioned on the graphic. Note that while macroscopic analysis of coprolites can provide evidence for parasites, in our case we did not identify any using that method.

In cases where human diet and health could not be inferred from human remains or coprolites, other animals that associated with humans in the region have been successfully used as a proxy^26,39,40^. Dogs are a promising surrogate, as they often live in close association with humans, which makes it likely that they had a similar diet and were impacted by similar parasites. Stable isotope values of dog and human bone collagen and tooth enamel have commonly been shown to be similar at the same archaeological site^41,42^, indicating that dogs and humans often have similar diets, and dog stable isotope measures have been used to infer human diet in regions where human remains are unavailable^39,43,44^. Similarly, dog coprolites can likely be used to infer the diet and health status of humans in the absence of human coprolites. Here, we analyze eleven dog coprolites from the region known as the American Bottom, the Mississippi River flood plain located in Southern Illinois. We use macroanalyses, DNA shotgun sequencing, and stable carbon and nitrogen isotopic analysis to provide insight on the diet and health of dog and human populations during the Late Woodland and Terminal Late Woodland periods, approximately 600–1050 AD (Fig. 1).

### Late Woodland diet in the American Bottom

During the Late Woodland period, (ca. 600–900 AD) humans living in the American Bottom region ate a varied diet that included both cultivated and domesticated crop plants and wild plants, as well as terrestrial and aquatic animal species (Supplemental Table 1). The cultivated crop complex is collectively known as the Eastern Agricultural Complex^45^, and comprised five domesticated plants: goosefoot (*Chenopodium berlandieri*), erect knotweed (*Polygonum erectum*), sunflower (*Helianthus annuus*), sumpweed (*Iva annua*), and native pepo squashes (*Cucurbita pepo* spp. *ovifera*)^45–48^. Little barley (*Hordeum pusillum)* and maygrass (*Phalaris caroliniana)* were also cultivated, though it is unclear whether they were domesticated^46,47^. These crops were supplemented with wild plants, in particular the nut taxa, hickory (*Carya* spp.), oak (*Quercus* spp.*)*, and black walnut (*Juglans nigra*), as well as wild fruits and greens^46^. Among the most important animals consumed were deer, fish, and seasonal waterfowl^49,50^.

The end of the Late Woodland period in the American Bottom is marked by a number of lifestyle changes, including an increase in population density and village size, and the rise of Cahokia, a large city with far-reaching cultural influences^51–53^. A shift in diet also occurred, with the introduction of maize during the Terminal Late Woodland Period (TLW, 900–1050 AD). Many of the native crops continued to be cultivated alongside maize during this time and the following Mississippian period (ca. 1050–1300 AD). However, while maize consumption accelerated rapidly after its initial introduction and continued into historic times, cultivation of most native crops declined and they eventually fell out of use^54^.

Multiple studies have explored the archaeobotanical and faunal remains from sites in the American Bottom, but few human remains have been recovered or analyzed from this time period (the Late Woodland and Terminal Late Woodland periods) in this region. While the coprolites have not been directly dated, the contexts they were recovered from suggest they date to these periods. Therefore, the coprolites of domestic dogs that we analyze in this study can be examined to provide additional insights into human diet and health. This analysis can confirm whether plant and animal remains recovered from Late Woodland and Terminal Late Woodland sites reflect human diet, and provide insights as to specific foods that were eaten, complementing the broad-scale view of diet that stable isotopes provide. Preservation of parasites or pathogenic microbes in these coprolites can also allow us to infer the health of the humans who interacted with the dogs that produced the coprolites. Coprolites have not been recovered from Late Woodland sites in this region, with the exception of a single site, Janey B. Goode, which has an abundance of dog coprolites^55^. At least 50 dog burials, skeletal remains of over 100 individual dogs, and 150 coprolites were recovered from Janey B. Goode^55,56^. The domestic dog was an important part of human communities in the American Bottom during the Late Woodland and Terminal Late Woodland periods, and dog burials have been identified at several archaeological sites in the region^56,57^. Given the close commensal relationship between dogs and humans, and congruent stable isotope analyses of dog and human collagen and enamel that demonstrate that dogs are a promising proxy for human diet in this region^39,41,43,58^, dog coprolites can be used to indirectly examine the diet and health of Late Woodland peoples in the American Bottom. Combining multiple lines of evidence, such as DNA, macrofossils and stable isotopes (^13^C, ^15^N), can provide a more detailed reconstruction of diet than is possible using only one method. By integrating existing and novel genomic and archaeometric methods (Fig. 1), we reconstruct aspects of the diet and health status of Late Woodland dogs, and use these data as a proxy for inferring the diet and health of contemporaneous human populations in this region.

## Results

### Coprolite species identification

Archaeological excavations at the Janey B. Goode site identified at least 50 dog burials^56^, recovered 150 coprolites and 5,400 skeletal parts representing more than 100 individual dogs^55^. In order to verify that the coprolites were canine in origin, we compared multiple lines of evidence obtained from our mixed-method approach. Many of the coprolites had white interiors, which reflects the consumption of bone and suggests that they are dog and not human. However, macroscopic remains in the coprolites can be inconclusive in identifying the source of the sample^59^. We used SourceTracker^60^, a Bayesian algorithm for estimating sources of microbes in a target community, to compare the coprolite microbiomes to a previously published non-Western shotgun human fecal microbiome^61^, as well as the dog microbiomes sequenced for this study. JBG 750-16 was the only sample that had a non-zero percentage match to the human fecal microbiome at 3%, though it also matched to the modern dog fecal microbiome at 43% (Supplemental Fig. 1). Two other coprolites matched the modern dogs at significant fractions (38% and 37%), while another matched at 3%.

### Macroscopic analyses

Animal bone and fish scale fragments were recognized in eight of the 10 coprolites from Janey B. Goode; none were found in the single coprolite from the East St. Louis site (Supplementary Table 2). Many of the bone and scale fragments were less than 0.5 mm in maximum diameter and could not be specifically identified. Six of the 11 coprolites contained fish bones, and one had several articulated fish vertebrae. Among the identifiable specimens, fish remains were most common with gar (*Lepisosteus* sp.) scales and bullhead (*Ameirus* sp.) bones recognized. Most of the fish bones, however, could not be identified to species. Bird (Aves) bone fragments were also identified, but they also could not be identified to species. The macrofaunal composition of the eight coprolites is similar to that of an earlier analysis of Janey B. Goode coprolites, in which fish remains predominate^55^. Taxa recognized in the previous analysis include gar, bullhead, bowfin (*Amia calva*), catfish family (Ictaluridae), frog/toad (Amphibia), rodent (Rodentia), and indeterminate fish, mammal, and bird^55^. No botanical material was identified in any coprolite.

### Dietary DNA analysis

Nine of the eleven coprolites yielded DNA. Metagenomic sequencing data from these nine coprolites were analyzed for dietary and microbial taxa (Supplementary Table 3). A total of 24 genera were identified as possible dietary components (Table 1, Supplemental Table 4), including fish (sunfish, bass, and gar), birds (ducks and geese), and frogs, as well as plants known to be utilized by Mississippian or Late Woodland peoples (grapes, walnuts, and tobacco). An intestinal parasite, *Toxocara canis*, was also identified in two coprolites.

**Table 1:** A list of the taxa recovered from DNA sequencing of the coprolites.

Scientific name	Common name	# Reads (average per coprolite)	# Coprolites	Found locally?	Found in archaeological record?
*Lepisosteus*	Gar	13 (13)	1	Yes	Yes
*Lepomis*	Freshwater Sunfish	6 (6)	1	Yes	Yes
*Micropterus*	Bass	2 (2)	1	Yes	Yes
*Perca*	Perch	2 (2)	1	Yes	Yes
*Ictalurus*	Freshwater Catfish	3 (1)	3	Yes	Yes
*Cyprinus*	Carp	4 (1.33)	3	Part of taxonomic family which includes native taxa	No*
*Homo*	Human	640 (71.11)	9	Yes	Yes
*Canis*	Dog	1508 (188.5)	8	Yes	Yes
*Anas*	Dabbling Duck	2 (2)	1	Yes	Yes
*Anser*	Grey Goose	6 (6)	1	Closely-related to native taxa (*Branta* and *Chen*)	No*
*Rana*	Frog	14 (4.67)	3	Closely-related to native taxa (*Lithobates*)	Yes
*Toxocara*	Intestinal Parasite	425 (212.5)	2	Yes	N/A
*Pristionchus*	Nematodes associated with insects	11 (11)	1	Yes	N/A
*Thelazia*	Eyeworms	2 (2)	1	Yes	N/A
*Xiphinema*	Root Nematodes	2 (2)	1	Yes	N/A
*Dendroctonus*	Bark Beetle	3 (3)	1	Yes	No
*Heteromita*	Flagellates present in soil	4 (4)	1	Yes	N/A
*Ipomoea*	Morning Glory	2 (2)	1	Yes	Yes
*Nicotiana*	Tobacco	4 (2)	2	Yes	Yes
*Hyoscyamus*	Henbane	2 (1)	2	Yes	No*
*Glycine*	Soybeans	9 (4.5)	2	Closely-related to native taxa *(Strophostyles)*	No*

In the reads column, the average number of reads per coprolite that contained reads from that species is noted in parentheses. Counts of reads per coprolite can be found in Supplemental Table 4. * indicates that the genus identified in the database is not present in the archaeological record of the American Bottom, but another genus in the same family is present. N/A: Studies of parasites have not been conducted in American Bottom archaeological sites.

No genus-specific dietary taxa were identified from the soil controls. The only genera that were identified in both the coprolites and the dog bone controls were *Homo*, *Canis*, and *Solanum* sp. (nightshade). Nightshade (Solanaceae) species native to the region include *S. ptycanthum* (West Indian/eastern black nightshade) and *Physalis virginiana* (ground cherry). Eastern black nightshade seeds have been identified in the archaeobotanical assemblages from both the Janey B. Goode and East Saint Louis sites. *Solanum* is poisonous, but the edible ripe berries of *Physalis* were probably consumed. Tobacco (*Nicotiana* sp., probably *rustica*) also belongs to the Solanaceae family. Although not considered edible, tobacco was certainly used by pre-Columbian people in the region, and dogs could have chewed on or even eaten the plants. Nonetheless, though edible taxa were present, *Solanum* has been previously identified as a contaminant in other plant aDNA studies. Therefore its presence as a contaminant in these coprolites cannot be ruled out^27,62,63^.

Nearly all other taxa identified are native to the American Bottom, with the exceptions of *Triticum* (wheat), *Glycine* (soybeans) and *Cyprinus* (Asian carp), which are now abundant in this region. It is likely that these taxa represent modern contaminants, though none were identified in any of the controls. These genera are closely-related to native species: *Triticum* is related to *Hordeum* (little barley) while *Glycine* is related to *Strophostyles helvola* (wild bean) and *Cyprinus* is related to several fish species that are native to the region. None of the native plant taxa and few native fish taxa have genomes represented in the Genbank nt (nucleotide) database, which would explain why they matched with closely-related but non-native taxa. Other taxa are rarely found in the region but are close relatives to more common native taxa—this is true of *Anser* (goose) and *Rana* (frog), which are closely related to *Branta/Chen* and *Lithobates* spp.*,* respectively.

### Microbial analysis

**Figure 2:**
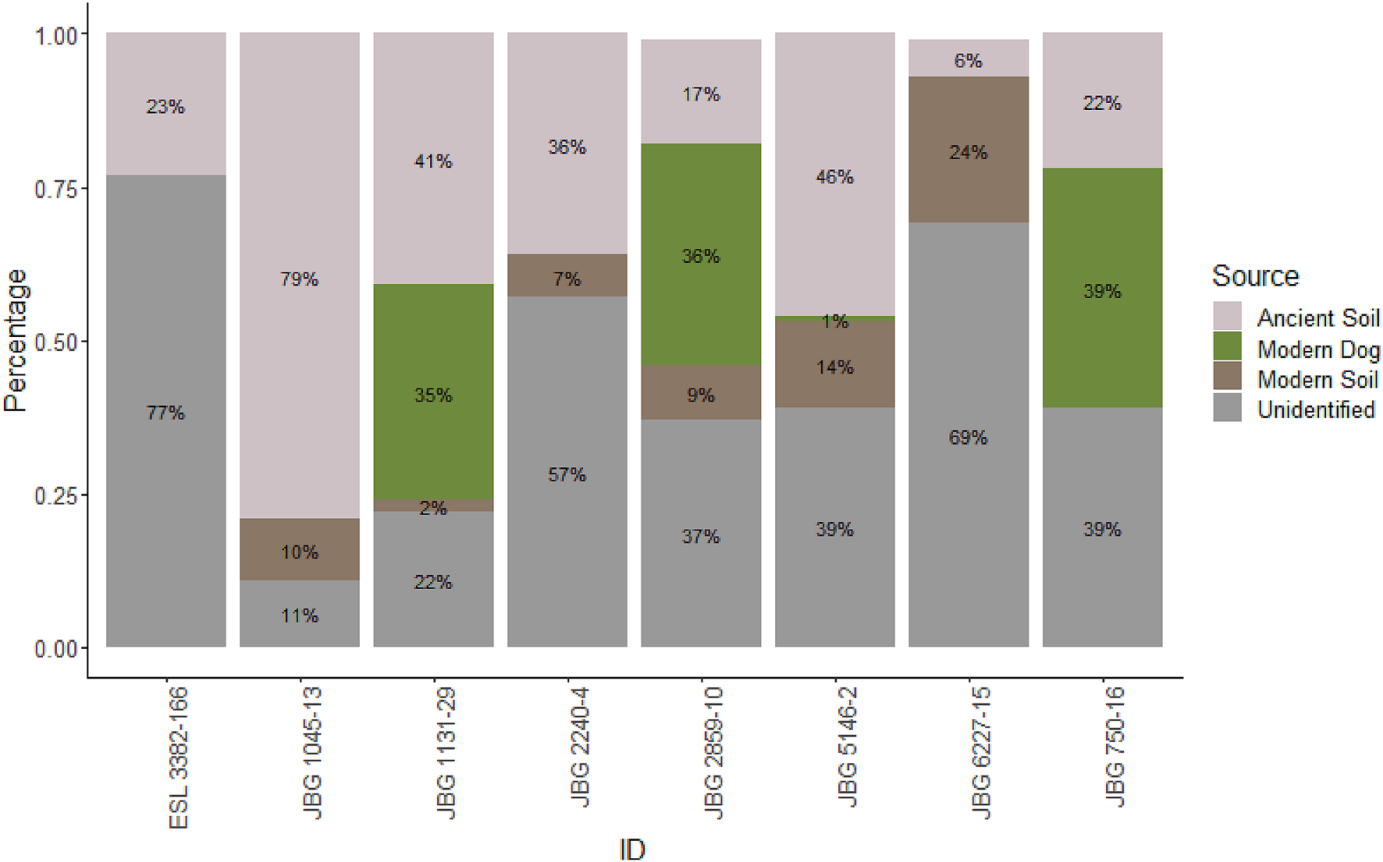
SourceTracker results, indicating soil sources and matches to modern fecal microbiome in the coprolites. The unidentified portions represent reads that could not be assigned to one of the listed sources (soil contaminants and the modern dog microbiome). This plot was created in R v. 4.0.2^111^ using ggplot2 version 3.3.2^112^.

The composition of microbial DNA recovered from the eight coprolites was compared to that of the soil and dog bone controls from Janey B. Goode, and two types of modern dog fecal microbiomes (from dogs fed low-protein and high-protein diets, respectively). Coprolite microbial composition was analyzed in SourceTracker^60^ using the modern dog fecal microbiomes, soil microbiomes, and microbes associated with the dog bone from Janey B. Goode as potential sources to help distinguish between fecal microbiome taxa and contaminating bacterial species (Fig. 2). This analysis indicated that only three of the coprolites contained microbes consistent with a dog fecal microbiome, while the remaining coprolites mostly contain soil microbes. Approximately one third of the reads from each of these coprolites were associated with the modern fecal microbiome, and the rest were split between soil-associated, bone-associated, and unidentified microbes that did not match potential sources.

**Figure 3:**
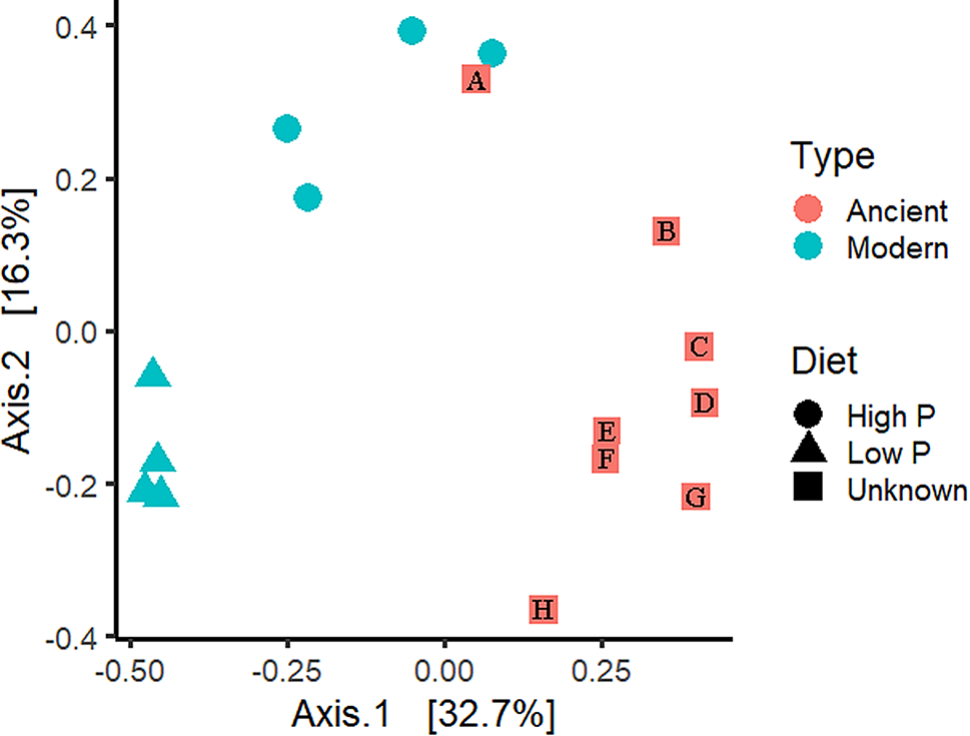
Principal coordinate analysis with filtered data showing spread of variation in the dataset. Three clusters are identifiable, and the groupings were verified by PERMANOVA (*p* = 0.00039). Only one coprolite sample (A—JBG 750-16) groups with the modern dog feces on the high protein diet. B refers to JBG 1131-29, C to JBG 1045-13, D to JBG 2859-10, E to JBG 6227-15, F to ESL 3382-166, and H to JBG 5146-2. This plot was created in R v. 4.0.2^111^ using ggplot2 version 3.3.2^112^.

**Figure 4:**
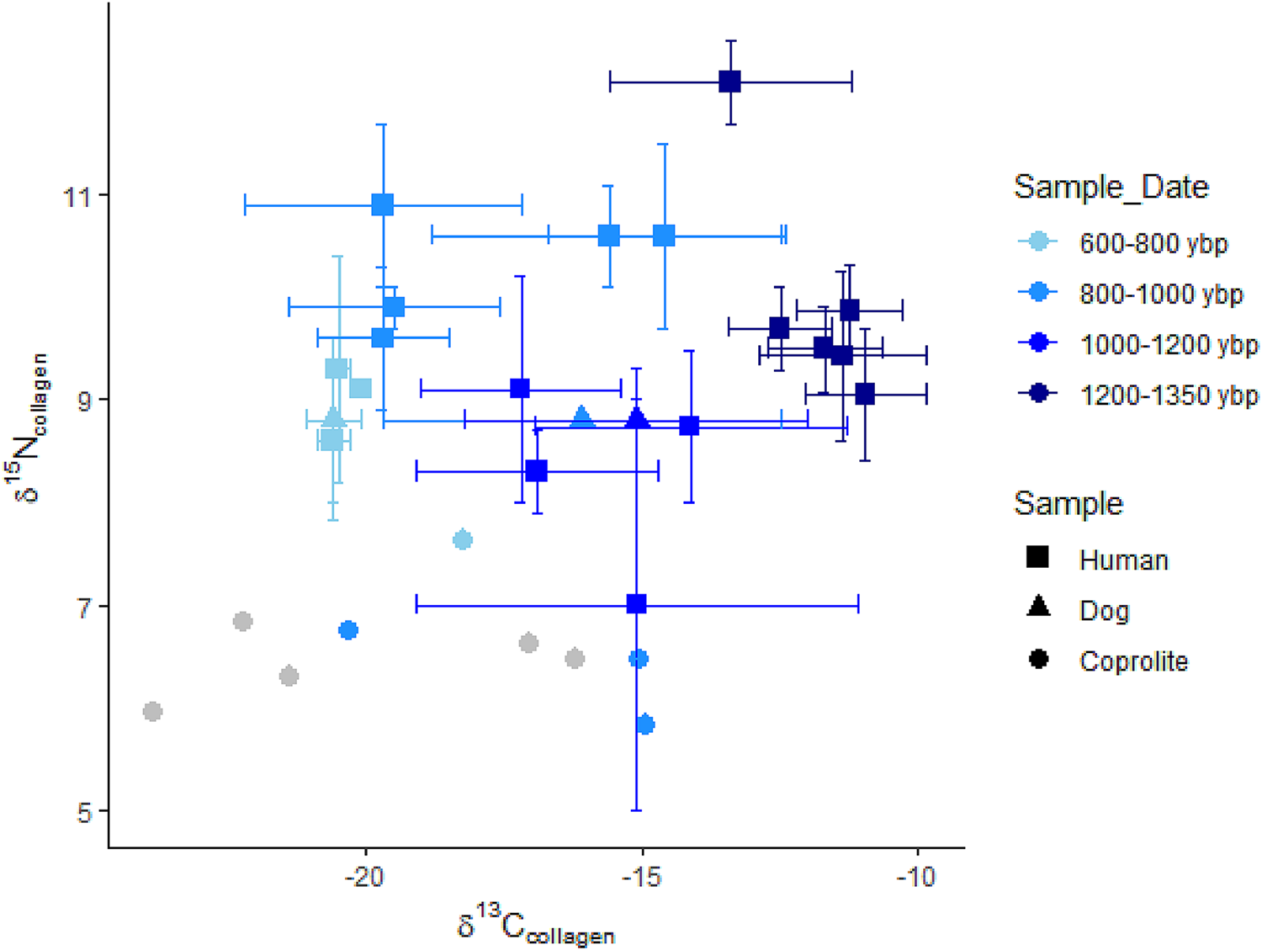
Coprolite δ^15^N and δ^13^C values, plotted in comparison to human and dog bone collagen values from Late Woodland and Mississippian populations in the American Bottom (data from Supplemental Table 6). Gray samples have an indeterminate age. Points lacking error bars reflect single individuals rather than populations. All coprolites are plotted individually (data from Table 2). This plot was created in R v. 4.0.2^111^ using ggplot2 version 3.3.2^112^.

Principal Coordinate Analysis (PCA) revealed distinct clustering between the ancient and modern dog fecal samples, with the exception of a single coprolite, JBG 750-16, which had both the highest portion of modern-associated dog fecal microbes and the largest number of microbial reads from coprolites in this study (Fig. 3). A PERMANOVA verified the difference between these clusters (*p* = 0.0004). To identify microbes that were differentially present in the modern and ancient samples, LEfSe (Linear discriminant analysis Effect Size) was applied to the eight modern samples and eight coprolites. When microbial taxa were used to discriminate between groups as represented in the PCA (Fig. 4), the high-protein cluster, including the single coprolite, was separated by the taxa *Bacteroides vulgatus*, *Escherichia coli*, and an unclassified species of *Turicibacter* (Supplementary Fig. 2). The modern and ancient clusters were separated by numerous taxa at various taxonomic levels, including a variety of putative soil microbes in the ancient cluster, and known gut microbiome inhabitants in the modern cluster. Methanogenic Archaea were solely present in the coprolite cluster.

When separating samples by diet, which places all coprolites in a single group, the modern dogs fed the high-protein diet were chiefly separated by *Bacteroides vulgatus*, the unclassified *Turicibacter* species, and *Helicobacter bilis* (Supplementary Fig. 3). Dogs fed the low-protein diet displayed a greater microbial diversity, including more variety in significantly different Proteobacteria species. This separation continued to highlight the abundance of putative soil microbes in the coprolites, as well as potential pathogens, such as *Enterococcus faecium, E. hirae,* and *Clostridium sordellii*. While functional content was assigned to each of the coprolite and fecal samples, no unique or discriminatory features were noted using the above methods.

The HOPS (Heuristic Operations for Pathogen Screening) pipeline^64^ screened all of the coprolites for putative pathogen reads, identifying damage patterns and fragment sizes to verify the ancient signature of these reads. Putative pathogens were selected based on potential for pathogenic behavior in modern contexts, as well as their absence in the healthy dogs in this study. These results highlighted two microbes that passed the criteria for aDNA: *C. sordellii* and *E. faecium* (Supplementary Table 5). The former was highly represented across all samples, while the latter had over a hundred reads in only a few coprolites, which were the same coprolites that had matches to modern fecal microbiomes in the SourceTracker analysis. After removing DNA reads that lacked the signature damage patterns associated with ancient DNA, *C. sordellii* was identified as an ancient taxa in two coprolites (Supplementary Fig. 4), while *E. faecium* was confirmed as ancient in all four coprolites that had a high abundance of those reads (Supplementary Fig. 4), including two that were shown to have a microbial signature consistent with a dog fecal microbiome.

### Coprolite carbon and nitrogen isotopes

Coprolites were also analyzed for their organic carbon and nitrogen concentrations and stable isotopic composition (δ^15^N and δ^13^C) in order to infer diet. Samples were ground and split into two sieved size fractions, which were analyzed separately to determine if particle size had an impact on elemental and isotopic composition. The coprolites had very low organic matter contents, with low nitrogen and carbon concentrations (weight %; < 0.01–1.25% N and 0.04–4.9% C) and highly variable atomic C:N ratios (4.01–13.90) (Table 2). Samples with less than 1% C are likely to be almost entirely composed of inorganic matter. Four coprolites (JBG 1045-13, JBG 2240-4, JBG 2859-10, and JBG 6553-2) had less than 1% C in both size fractions, so their stable isotope values were not used to infer diet. Standards used in the analysis, as well as re-runs of individual samples, were all consistent for both δ^13^C and δ^15^N and wt% C and N. Size fractions of the same sample with sufficient carbon concentrations (> 1%) show replicate δ^13^C and δ^15^N values that differ by less than 1.0‰. The large fraction of these samples also had higher wt% C. When coprolites with low organic C and N concentrations are removed from the analysis, samples with high organic C concentrations have average δ^13^C and δ^15^N of − 18.93 ± 3.23‰ and 6.53 ± 0.53‰, respectively (Table 2). Large size fractions of these samples have higher C and N concentrations than their small fractions (0.89% and 0.24%, respectively) suggesting more dietary organic content and less inorganic sediment in the large fraction.

**Table 2:** Coprolite elemental and isotopic composition.

Coprolite catalog no	Site	Size Fraction	δ^15^N	Wt % N	δ^13^C	Wt % C	Atomic C:N	Diet % C_4_
JBG 750-16	Janey B. Goode	Large	5.822	0.30	− 15.121	2.65	10.23	69.9
		Small	6.461	0.20	− 15.218	2.29	13.16	69.2
JBG 1045-13	Janey B. Goode	Large	5.743	0.07	− 23.010	**0.27**	4.32	17.3
		Small	6.291	0.08	− 23.253	**0.44**	6.52	15.7
JBG 1131-29	Janey B. Goode	Large	6.735	0.23	− 20.401	1.19	5.95	34.7
		Small	6.103	0.08	− 22.444	**0.38**	5.39	21.1
JBG 2240-4	Janey B. Goode	Large	6.893	0.02	− 26.876	**0.10**	6.92	− 8.5
		Small	10.229	0.01	− 25.581	**0.08**	6.00	0.1
		Small (rerun)	10.701	0.02	− 24.726	**0.05**	3.96	5.8
JBG 2859-10	Janey B. Goode	Large	5.848	0.06	− 23.210	**0.30**	5.54	15.9
		Small	7.689	0.07	− 23.652	**0.51**	8.41	13.0
JBG 5146-2	Janey B. Goode	Small	5.945	0.27	− 23.921	1.14	4.94	11.2
JBG 6227-15	Janey B. Goode	Large	7.621	0.15	− 18.160	1.82	14.26	49.6
		Large (rerun)	7.622	0.16	− 18.376	1.89	13.55	48.3
		Small	6.265	0.07	− 23.897	**0.40**	6.52	11.3
JBG 6553-2	Janey B. Goode	Large	N/A*	N/A*	− 26.876	**0.10**	N/D*	− 8.5
		Large (rerun)	N/A*	N/A*	− 25.581	**0.08**	N/D*	0.1
		Small	N/A*	N/A*	− 24.726	**0.05**	N/D*	5.8
JBG 7186-2	Janey B. Goode	Large	6.304	0.33	− 21.489	1.14	4.01	27.4
		Small	6.826	0.25	− 22.323	1.32	6.22	21.9
JBG 7298-2	Janey B. Goode	Large	6.617	1.26	− 17.180	4.91	4.56	56.1
		Small	6.470	0.55	− 16.382	3.02	6.41	61.5
ESL 166	East St. Louis	NA*	NA*	NA*	NA*	NA*	NA*	NA*

δ^15^N and δ^13^C values are listed for the large and small fractions of each coprolite, as well as weight percent N and C and the atomic C:N ratio. N/D* indicates that the nitrogen concentration was below peak detection limits, so C:N could not be calculated. Only the small fraction for 5146-2 was available for analysis. Samples with less than 1% C are indicated in bold font. Diet % C_4_ is calculated assuming δ^13^C end member values of − 26.5‰ and − 11.5‰ for 0% and 100% C_4_, respectively, and a diet-feces difference of − 0.9‰. NA* indicates that the coprolite sample from East St. Louis was not available for stable isotope analyses.

Previous research shows that fecal δ^13^C differs from diet δ^13^C by − 0.9‰ and δ^15^N differs by + 1.0‰^65^. This is a much smaller offset compared to that between collagen and diet, which is estimated to be + 5‰ for δ^13^C^66–68^ and + 3–4‰ for δ^15^N^69^. When coprolite isotope values are adjusted to be directly comparable to collagen isotope values, coprolites generally have δ^15^N values slightly lower than those of Terminal Late Woodland humans (Supplemental Table 6). Ancient American Bottom human bone collagen average δ^13^C values range from − 20.6 to − 11.0‰ (Supplemental Table 6, Fig. 4) reflecting diets with 5% to 70% C_4_-based diets. Maize and maygrass are the only C_4_ plants preserved in archaeobotanical assemblages. Dog collagen δ^13^C values in this region range from − 20.6 to − 15.1‰, reflecting diets with 5% to 40% C_4_ (Supplemental Table 6). Well-preserved dog coprolites δ^13^C values reflect diets with approximately 10–70% C_4_-based foods.

## Discussion

The coprolites from Janey B. Goode were subjected to three types of analyses, including macroscopic identification of faunal and botanical remains, DNA sequencing to identify dietary and microbial taxa, and stable carbon and nitrogen isotope analysis to identify broad trends in diet. These varied methods also permit assessment of coprolite preservation. We found that the recovery of dietary and putative gut microbial DNA, faunal remains and C and N isotopes were all correlated. Nearly all the coprolites with carbon concentrations below 1% by weight also contained contamination in the form of soil microbes, confirming that the isotope results for these samples would likely not reflect diet. Moreover, samples with low C concentrations also had low δ^13^C values, likely reflecting soil organic carbon derived mainly from C_3_ plants. Similarly, the coprolites with the lowest number of dietary reads produced one of the larger number of microbial hits, most of which are soil-associated. Conversely, the three coprolites with the highest similarity of microbes to modern dog gut microbiomes all had average or above-average taxonomic reads, and two of the three had high-quality stable isotope results, consistent with good preservation.

Faunal identification, DNA sequencing, and stable isotope results complement each other and collectively provide a detailed view of ancient dog diet in the American Bottom. Faunal and dietary DNA analyses generally show agreement, with fish taxa identified in five coprolites through both analysis methods, and specific genera (*Lepisosteus* and *Ameirus/Ictalurus*) identified with both methods in two coprolites (Table 1, Supplementary Table 2). Low nitrogen and high carbon isotope values may also reflect a diet high in fish, as ancient fish samples from the nearby Illinois River, including some that the dogs are known to have eaten (*Perca, Micropterus*, and *Lepomis*), show isotopic values that are consistent with the ones calculated from the coprolites^70–72^. Additional analyses of ancient fish from the Mississippi River would be necessary to fully distinguish between a diet of C_4_ plants and a diet of fish with high δ^13^C. Supporting evidence for a diet high in fish is also found in the best-preserved coprolite (JBG 750-16), whose microbiome was most similar to those of modern dogs, and clustered with the high-protein diet group (Fig. 3). Fish have been previously reported as a dietary mainstay in multiple dog populations, including Archaic Period dogs recovered from the American Bottom^73^. This work expands on the analyses performed by Fortier et al.^55^, and many of the taxa identified in his macroscopic analysis (including *Lepisosteus*) were also found in the coprolites analyzed here. Some taxa identified in Fortier et al. (such as specific mammals) were not identified in this study, reflecting dietary variability among Late Woodland and Terminal Late Woodland dogs.

Coprolites have lower δ^15^N and higher δ^13^C values relative to those of collagen and apatite of human and dog Late Woodland and Terminal Late Woodland populations^54,74–77^ (Supplemental Table 6), and also vary significantly between samples. The difference in stable isotope values between sample types is likely due to the fractionation of the dietary components: collagen reflects the isotopic composition of mainly the protein source, with δ^13^C consistently enriched by 5‰ and δ^15^N by 3–4‰ relative to diet^66,67^, while coprolites approximate the isotopic composition of whole diet, with δ^13^C depleted by ~ 0.9‰ and δ^15^N by 1‰ relative to diet^13^. Variation in isotopic composition for the individual coprolites likely reflect a combination of short-term changes in diet and proportions of diet to soil organic matter. Temporal changes in diet may also be reflected here; contemporaneity among coprolites has not been confirmed, as they have not been directly dated.

The dogs at Janey B. Goode had a varied diet of fish, waterfowl, and local plants. Many taxa were identified using DNA sequencing, including grapes, walnuts, possible little barley, and waterfowl. These species were previously identified from Late Woodland and Terminal Late Woodland archaeological sites (Supplemental Table 1). Stable isotope results from the coprolites suggest a diet of terrestrial fauna, fish with low δ^15^N and high δ^13^C values, and C_3_ plants with the possible addition of C_4_ plants such as maize. Maize is absent from Late Woodland sites in the region and is only identified from Terminal Late Woodland contexts (post-dating AD 900^46,54,78–80^), and maize was not identified in the DNA reads recovered from the coprolites. Explanations for the latter include: coprolite deposition preceded the introduction of maize (ca pre AD 900^54^); the meals reflected did not include maize; or that maize DNA was too degraded to identify. Evidence suggests that maize was processed with nixtamalization and/or heating processes in the American Bottom^54^, and both result in fragmented DNA^81^, which would likely be further degraded as a result of digestion and decomposition in the soil. Deer, which were commonly consumed by humans, was not identified via macroanalyses or DNA sequencing which, paired with a scarcity of carnivore-gnawed deer bone from Janey B. Goode, suggests that dogs were not regularly provisioned with deer. Given that coprolites likely reflect only a few meals, seasonal variation in availability of some foods may also explain their absence from these samples.

Multiple sources of evidence in these coprolites point to markers of health among individuals in this canid population. First, the presence of *Toxocara canis* DNA in the best-preserved coprolite highlights a possible infection in that individual. Second, this same coprolite contains a number of DNA reads with an ancient signature mapping to *E. faecium*, a known putative pathogen that can cause a variety of infections (Supplementary Table 5). Both *T. canis* and *E. faecium* can be transmitted through contact with the feces of an infected individual, for example via contaminated drinking water^66^. Problems with public health are often associated with urbanization and overcrowding in the archaeological record^82^, which would be consistent with an increase in community nucleation during the Terminal Late Woodland period^83^.

Our findings regarding diet and health status in dogs can also be used to infer the same in the humans who lived at Janey B. Goode. Humans and dogs in the American Bottom during the Late Woodland period have been shown to have similar diets, based on collagen and enamel isotopic analyses^54^, suggesting that the humans living at Janey B. Goode consumed a mix of fish, waterfowl, and plants, similar to the dogs. However, as dogs often scavenge the remnants of human meals^41,44,84^, humans may have consumed additional foods, such as deer, that dogs were not given access to. If the elevated δ^13^C values from some dog coprolites represent maize, humans were likely consuming maize as well, consistent with patterns from across the American Bottom^46,54,79^. Although we cannot confirm whether the humans at Janey B. Goode were impacted by the pathogens and parasites that the dogs carried, it is reasonable to infer that they were, given that parasites have been found to be widespread in many ancient human populations^85,86^, and given the archaeological evidence that suggests dogs and humans at Janey B. Goode were in close contact^87^. As other studies have noted, a shared diet and environment between companion animals and humans also increases the likelihood of infection by similar parasites and pathogens^5,6,37,85,88^.This suggests that humans living in the American Bottom during the Late Woodland to Mississippian periods were also impacted by conditions associated with urbanization. Dogs were an important part of life in the American Bottom during the Late Woodland period, and their coprolites are informative not only about their own diet and health, but that of their human companions as well.

In a modern microbiome, the presence of these pathogens might coincide with other shifts in the microbiome, including the rise of other pathogenic species, as the health of the system deteriorates. We cannot make a similar determination in this case, given the incomplete reconstruction of the coprolite microbiomes, as well as residual contamination. Another potential pathogen present in all coprolites is *C. sordellii,* which has been implicated in gastroenteric disease^89^. Despite the high overall abundance in the coprolites, only two samples bore reads with markers consistent with aDNA damage. The spore-forming ability of this microbe may have biased its survival and recovery, leading to the observed increase in relative abundance. Therefore, while a potential pathogen, it is also possible that these *C. sordellii* reads reflect a uniform contaminant in the coprolites.

Previous interdisciplinary approaches to analyzing coprolites have relied on many of the same methods applied here and have often noted similar challenges. In this study, the combination of methods has provided a more holistic analysis of coprolite contents, rather than relying on genetic material or macroscopic remains alone. By shotgun-sequencing all DNA in these coprolites, rather than focusing on a single hyper-variable region, we were able to recover the DNA from a number of taxa. Comparison of the stable isotope values to the individual dietary taxa identified provided further context to the diet of these individuals. On the health side, the presence of both parasites and pathogens together provides more compelling evidence for infection, particularly because these both appear in the same, well-preserved sample. Using HOPS to verify the ancient signature of the microbial pathogens in this coprolite is also novel, as most microbial coprolite studies to date have relied on methods such as SourceTracker to highlight contamination and verify the authenticity of microbial reads.

DNA recovery from the coprolites may have been impacted by our extraction method. We used a stool DNA extraction kit to take advantage of the inhibition removal techniques included in those workflows, and modified the methods to improve the recovery of degraded DNA (see Methods below). However, recent work suggests that traditional ancient DNA extraction methods, such as the Qiagen Minelute kit, work best for recovering DNA from coprolites^90^. Therefore our DNA yield may have been higher if we had used a different extraction method.

As with other ancient samples, limited preservation of DNA and organic material in coprolites makes them incomplete records of diet and health. A fecal microbiome recovered from coprolites is incomplete^59^, which makes it difficult to compare to modern fecal microbiomes. This is due to numerous changes over time, including the transition from an anaerobic environment in the distal gut to the outside aerobic environment, which favors the survival of some microbes over others, thus leading to shifts in microbial proportions over time^32^. For example, *C. sordellii* showed great abundance in all coprolites, but its spore-forming abilities may have biased the abundance of DNA in the samples. The abundance of microbes matching with the high-protein modern dogs in the best-preserved coprolite may also be a result of this type of bias. Similarly, dietary material that is more likely to survive digestion is more likely to be successfully identified^59^. For example, *Lepisosteus* was one of the most common taxa identified, both from DNA and from macroscopic analyses, but this is more likely due to the hardness of their scales than to their abundance in diet. This is also demonstrated by the successful recovery of plant DNA from the majority of coprolites, despite the absence of visible plant material in the macroscopic analysis. While we removed visible bones and scales from the coprolite prior to DNA extraction or processing for stable isotope analysis to limit this bias, this may have also favored the recovery of DNA from taxa that were more thoroughly digested prior to excretion. Cooking may also play a role in whether a food type was preserved in the coprolites^81,91^, possibly biasing the results in favor of uncooked foods, whose DNA would not be degraded by heat prior to consumption. Stable carbon isotope data indicate consumption of C_4_-based foods, although none are represented in coprolite DNA or microfossils. Therefore isotopic analysis can be useful for identifying foods whose traces are not preserved in coprolites due to cooking or other forms of food processing such as grinding, digestion and decomposition.

Limitations of DNA databases can also make the identification of certain species difficult and bias results. Model organisms, domesticated species, and microbiomes sampled from individuals with industrialized diets are the most likely to be sequenced. Therefore, native species and novel microbes are less likely to be identified due to an inability to match them sufficiently to taxa in a database. This problem is further exacerbated by the accumulation of DNA damage in ancient sequences^92^, which makes it less likely that a DNA read recovered from coprolites would be an exact match to a sequenced modern taxon. Thus, a match to a single species does not necessarily indicate that the DNA sequence is from that species; the DNA may be from a species that has not yet been published in a genetic database but is a close relative (within the same genus or family) to others that have been published. This kind of misidentification is likely for several taxa that were identified, including *Cyprinus* and *Triticum*, that were not native to the American Bottom during the Late Woodland through Mississippian periods. This can also be seen in cases where taxonomy was unable to be verified past a certain level, as with Supplemental Fig. 2. The ability to identify taxa of interest is partially limited by read length, and therefore longer reads (> 100 bp) may allow for more specific taxonomic assignment. Genomic sequencing of additional species, such as native plant taxa, and under-represented microbiome diversity, would also help confirm the identity of the DNA reads recovered from coprolites, as well as enable the identification of additional taxa.

## Conclusions

This integrative analysis of coprolites demonstrates the value of using multiple methods to assess the diet, microbiome, and health of an individual from a single sample. Specific dietary components and parasites can be identified through DNA sequencing and macroscopic analyses, while stable isotope analysis can shed light on large-scale trends in diet (for example, whether or not maize was consumed). Analysis of microbes in coprolites can be helpful for reconstructing part of the fecal microbiome of ancient dogs, and can also be useful in highlighting large-scale dietary trends. Preservation was correlated across the analysis types, with successful dietary and pathogen taxa identification occurring more often in coprolites with greater microbial similarity to modern dog fecal microbiomes. By using multiple analyses, we demonstrate that the ancient dogs at Janey B. Goode were consuming a varied diet of fish, waterfowl, and numerous plant taxa. While maize was rare in the Terminal Late Woodland period^54^, dogs may have been consuming it as well, demonstrating the possibility of its presence in the region. We also gain a perspective on health, as at least one individual harbored both parasites and pathogens that indicate issues with public health. Humans and dogs at Janey B. Goode during the Late Woodland period likely had a similar diet, given the overlap between human and dog isotopic composition from other sites in the area, and they may have faced similar health risks. By using these analytical methods together, we gain a better view of diet, sample preservation, and the microbiome of the canine community at Janey B. Goode. The canine surrogacy approach used in this study may also provide insights into the diet and health of the human population.

## Methods

### Community collaboration

The Illinois State Archaeological Survey performed investigations at the Janey B. Goode site under the terms of a memorandum of understanding among the Federal Highway Administration, the Illinois Department of Transportation, and multiple Indigenous groups with ancestral ties to Illinois. The Janey B. Goode investigations were initiated prior to this memorandum being formally put into effect, but all associated funerary objects and ancestral remains are treated in accordance with the Illinois Human Skeletal Remains Protection and Repatriation Act (HSRPA) (ILCS 3440, 17 IAC 4170). We are currently in the process of formally presenting this work to these Indigenous communities.

### Samples

Ten coprolites used in this study are from the Janey B. Goode site (11S1232), located in Brooklyn, IL, eight kilometers from the main site of Cahokia^93^. Janey B. Goode was occupied from the Late Woodland through the Mississippian periods. Approximately 5400 skeletal parts of dogs were recovered, representing 103 individual dogs; 55 individuals were identified as coming from burial contexts, including burials and isolated elements within storage pits and depression structural features^87^. This may be the largest assemblage of archaeological dog remains in the United States^93^. The majority are associated with the Terminal Late Woodland (900–1050 AD) occupation of the site^57,87^. A small number of dogs are from the Late Woodland (650–900 AD) and Mississippian (1050–1300 AD) components. The condition of the dog burials range from complete individuals to isolated crania, with animals of all age groups represented. The isolated skull and jaw portions likely represent elements used in ritual events^56,57,87,93^. The paucity of dog burials during the Mississippian period can be attributed to increased consumption of dogs, likely as part of feasts or ceremonial dining, during the Mississippian period^57,93,94^.

A single dog coprolite out of 12 identified coprolites from the East Saint Louis site (11S706) was also included in this study. The East St. Louis precinct is the second largest civic-ceremonial complex and one of three primary precincts that, along with St. Louis and Cahokia, comprise Greater Cahokia^95^. The occupation of East St. Louis spans the Terminal Late Woodland through Mississippian periods. Skeletal elements representing 29 dogs were identified in the ESTL faunal assemblage. Most are associated with the Terminal Late Woodland II (n = 18) (ca 975–1050 AD) and Early Mississippian (n = 4) (ca 1050–1150 AD) periods. Additional elements attributed to domesticated dogs were present in features and /or from surface contexts for which temporal components could not be determined. As at Janey B. Goode, dog remains ranged from complete articulated burials to isolated cranial and sub-cranial elements. The analyzed coprolite was identified in Feature 3382, a massive pit features that dates to the Terminal Late Woodland II (ca 975–1050 AD) period.

Preservation of coprolites in the American Bottom is unique among open-air archaeological sites in eastern North America^55^. Massive amounts of limestone (28 tons), recovered at the site may have enhanced coprolite and bone preservation^93^. No special precautions were taken to limit contamination during excavation, but once the coprolites were identified as such, they were handled with gloves and were stored in separate sterile containers when not being handled^55^. The coprolites from Janey B. Goode and East St. Louis were initially identified as dog due to their macrofaunal contents, which include many bones and fish scales, and because they have white interiors, reflecting the consumption of large amounts of bone. A previous macroscopic analysis of six of the coprolites identified numerous taxa including rodents, birds, amphibians, and fish, as well as unidentifiable plant fibers^55^. Ten coprolites that were not previously analyzed were selected for analysis in the present study (Supplemental Table 2). Our workflow of analyses is summarized in Fig. 1.

For comparative purposes, eight fecal samples were obtained from four modern dogs, two from each individual. These dogs are beagles housed at the veterinary school at the University of Illinois at Urbana-Champaign. The beagles were initially fed a low-protein (LP) dry kibble diet (24.1% protein, 13.3% fat, and 9.6% fiber) for 28 days before the first fecal sample was collected. The dogs were then transitioned to a high-protein (HP) wet food diet (45.7% protein, 30.3% fat, 7.3% fiber) over the course of 9 days, with the proportion of wet food increasing every few days until, on day 10, the dogs were consuming entirely wet food. After two weeks on this high-protein diet, fecal samples were collected again. The dogs involved in the study were individually housed to prevent cross-contamination among individuals. These fecal samples were previously used in analyses to compare the impacts of different modern dog diets on nutrient digestibility and fecal microbiota^96^.

### Coprolite DNA extraction, library preparation and sequencing

All extraction steps involving DNA were performed in the Ancient DNA Laboratory at the Carl R. Woese Institute for Genomic Biology at the University of Illinois. DNA from ancient samples is degraded and fragmented, and is easily contaminated with DNA from modern samples^92^. The Ancient DNA Laboratory is physically separated from any lab where modern DNA samples are handled. In this lab, all workers wear full-body suits and multiple layers of gloves to prevent sample contamination, and all surfaces and tools are cleaned with bleach and DNA-Off before and after use, and a UV Cross-linker is used to sterilize all lab equipment. These precautions are taken to limit contamination as much as possible.

Coprolites were weighed prior to sampling. Using a scalpel, the end of the coprolite was sliced off, exposing the interior. A Dremel drill was used to drill the inside of the coprolite for 300–400 mg powder, which was extracted using the QIAamp DNA Mini Stool Kit following the protocol in Archie et al.^97^, with the following additional modifications designed to optimize degraded DNA recovery: the powder was dissolved in 800 uL EDTA and 300 uL N-lauryl sarcosine, tubes were vortexed for 5 min with the InhibitEx tablet, and the DNA was eluted using two 30 uL aliquots of AE buffer, with 30 min of incubation time for each aliquot. We added the EDTA and N-lauryl sarcosine to break down bone fragments in the coprolites, used a longer period of vortexing to ensure the coprolites were properly broken up, and used longer DNA elution incubation times to attempt to maximize DNA recovery. A dog bone control recovered from Janey B. Goode was co-extracted with one of the extractions, to screen for lab and environmental contamination. A second control, a dog bone extracted using a standard ancient DNA extraction method using a Qiagen PCR Purification kit (see^98^ for detailed methods), was used to differentiate between lab reagent contamination and environmental contamination. If a contaminant was found in either control, the identified taxa was removed from the analysis.

Bacteria in soil and in feces contain enzymes that can inhibit the polymerases in PCR, which is a common problem for ancient DNA samples^99,100^. The extracted coprolite DNA was tested for PCR inhibition by adding 2 µL of the coprolite extract to a PCR reaction using DNA extracted from ancient dog bones that has previously amplified successfully (1:1 dog bone DNA extract to coprolite DNA extract ratio), and dog mitochondrial DNA primers^98^. If the sample spiked with a coprolite extract failed to amplify (which would indicate PCR inhibition), the extract was run through a silica column using a Qiagen PCR Purification Kit. This step was repeated until the spiked control amplified successfully, which indicated that the majority of PCR inhibitors were removed. All coprolites that were submitted for sequencing took no more than two re-extractions to remove all inhibitors.

Whole genome shotgun DNA libraries were built from the extracts using the NEBNext Ultra DNA Library Prep Kit for Illumina. Amplification of library DNA fragments was performed in a laboratory designed for the extraction and amplification of modern DNA, the Malhi Molecular Anthropology Laboratory at the University of Illinois at Urbana-Champaign. The library was cleaned to remove adapters using Agencourt Ampure XP beads (with a 1:1 beads to library ratio), and samples were indexed using the NEBNext Multiplex Oligos for Illumina prior to amplification, which was done as recommended by the manufacturer. A second amplification was prepared from the amplified product using Phusion High Fidelity Master Mix with a reduced volume of primers (1.5 uL instead of 2.5), the addition of 1.5 uL of DMSO and 1 uL of BSA, with 5 uL of DNA, with four reactions prepared per sample. PCR thermocycling conditions followed the directions of the manufacturer, using the maximum time for the denaturing, annealing and extension steps and a 65 °C annealing temperature, for 12 cycles. The four reactions per sample were pooled and cleaned using a Qiagen Minelute PCR Purification Kit. The amplified libraries were visualized on an agarose gel and quantitated using a Qubit Fluorometer, then pooled and size-selected for 185–650 bp to exclude adapters. The pool was sequenced on two lanes of an Illumina HiSeq 4000 using 100 bp single-read chemistry, at the Roy J. Carver Biotechnology Center at the University of Illinois at Urbana-Champaign.

### Soil control extraction

To control for microbial contamination in the coprolites, soil from the excavation pits at Janey B. Goode was sequenced. The soil-associated DNA was extracted in a molecular lab space, in a pre-PCR area designated for follow-up procedures from the ancient lab, to further minimize risk of contamination. The soil extraction used the same protocols for DNA extraction, library prep, and sequencing that were applied to the coprolites to avoid biases in later comparisons.

### DNA extraction of fecal samples

DNA from modern fecal samples was extracted in a BL-2 approved lab dedicated to molecular biology research, with sterilization protocols prior to extraction, the use of sterile tubes and filtered pipette tips, and physical separation between DNA extraction and PCR amplification. The DNeasy PowerSoil Kit was used, following the provided protocol with no modifications. Libraries were constructed using KAPA Library Preparation Kits, and then pooled and shotgun-sequenced (100 bp single reads) on a single lane of an Illumina Hi-Seq 4000 at the Roy J. Carver Biotechnology Center at the University of Illinois at Urbana-Champaign.

### Taxonomic analysis

Once sequencing results were obtained, the sequencing reads were processed using a bioinformatics pipeline. Adapters were trimmed from the reads using the FASTX toolkit (http://hannonlab.cshl.edu/fastx_toolkit/index.html), and a hard trim of 3 base pairs (bp) and a soft quality trim was also applied to the reads. Only reads that were greater than 50 bp in length were retained for analysis. The reads were de-duplicated, and then assembled into longer contiguous reads (contigs) de novo using Abyss version 1.3.4 with a k-mer size (or match length) of 36^101^. This assembly of shorter reads into longer contigs was performed with the aim of being able to identify the source of the DNA with higher specificity. The assembled reads were then compared to the National Center for Biotechnology Information (NBCI) nt (nucleotide) database using BLAST + 2.6.0^102^. Results were filtered following the guidelines established in Warinner et al^103^. Reads that matched a taxon in the NCBI database had to be at least 75 bp in length, with 100% identity across the entire length of the read. Additionally, if multiple species matched a single read, all of the species that matched had to be part of the same genus. We retained all matches that belonged to animal or plant phyla as possible dietary components and removed any matches that were shared between samples and controls from further analyses.

The resulting reads were visualized using MEGAN6 Community Edition^104^. For each, the taxon had to be present in at least two reads across all samples analyzed and represent at least 0.1% of all reads. After the filtering steps, 32 genera remained. We then performed local BLAST searches, with the non-redundant nucleotide BLAST database using all reads that matched to a dietary taxa, to assess the likelihood that the taxa were correctly identified. If a sample had greater than 10 reads that matched to a specific taxon, we randomly selected 10 reads to analyze. If the read was found to match multiple genera or was a close match (> = 98% identity) to human, dog, or bacterial DNA, the read was discarded.

### Microbial analysis

The reads from the soil and modern fecal libraries were trimmed and quality filtered using KneadData^105^. The trimmed and quality filtered reads from the coprolites, soil, and modern fecal libraries were all run through Metaphlan2 to assign taxonomy, using default parameters. The resulting data was imported into R and phyloseq for downstream analysis^106^. First, the taxonomic data was parsed through SourceTracker (v1.0), a Bayesian algorithm designed to estimate partitions of sources in sample data^63^. This package was also used to verify the canine origin of the coprolites, in comparison to a non-Western human microbiome^60^, by running the same analysis with that human microbiome included. After using SourceTracker, the soil data was filtered out of the coprolites using KneadData^105^. Subsequently, the coprolites were also analyzed through PCA in phyloseq, as well as run through a linear discriminant analysis in the form of LEfSe in biobakery; the threshold for significance was increased to 4.5 based on permutation tests^105,106^. As shotgun data was available, humann2 was used to assign functional groups to the coprolite and fecal datasets^105^*.* PERMANOVA was performed using the vegan package in R^107^. The HOPS pipeline was used to verify the authenticity of aDNA for select microbes in the coprolites^64^. In brief, a MALT database was constructed using genomes from the latest NCBI RefSeq archive. The default configuration was used for HOPS, with the specification of only using the ancient results.

### Stable carbon and nitrogen isotope analysis

Nine coprolites were selected for isotopic analysis. After each coprolite was sampled for DNA analyses, it was then prepared for isotope analysis. Part of the interior of the coprolite was excised using a scalpel, to ensure that surface contaminants were not sampled, and any visible bones or scales were removed prior to grinding. The coprolite was then ground in a mortar and pestle and was separated into two size fractions: 117–250 µm and < 117 µm, using geological sieves. Roughly 600 mg of each coprolite and size fraction were weighed out into annealed glass 50 ml centrifuge tubes. Approximately 20 mL of 0.2 M HCl was added to each tube and vortexed to thoroughly disseminate the sample, demineralize bone and remove sedimentary and diagenetic carbonates. Tubes were shaken for 1 h at 200 rpm and left to react overnight. The next day, 0.2 M HCl was replaced, and shaking and overnight reactions were repeated. The sample was centrifuged and rinsed 4 times with distilled water to neutrality. Then, 20 mL of 0.125 M NaOH was added to remove base-soluble soil organic contaminants. The samples were shaken at 200 rpm for 12 h, and then rinsed 4 times to neutrality. Samples were dried in a 70 °C oven for 12 h. Dry samples were weighed and percent weight loss was calculated.

Isotopic analyses were performed at the Illinois State Geological Survey, Prairie Research Institute, at the University of Illinois at Urbana-Champaign. Each dried treated coprolite sample was homogenized, and a minimum of 8000 µg of powdered coprolite was weighed into a tin foil capsule for analysis. The Carlo-Erba NC-2500 Elemental Analyzer was used to convert the organic matter into purified N_2_ and CO_2_ for isotopic analysis. A total of 34 samples in compressed tin capsules, including three samples that were selected randomly as replicates, were placed in the Elemental Analyzer carousel along with 14 samples of standards of thiourea, L-serine and hydroxyl-L-proline. The elemental analyzer converts organic matter into purified N_2_ and CO_2_ by combustion, oxidation and reduction at high temperatures, and transfers these gases in a helium carrier gas controlled by a Thermo-Finnegan ConFlo IV device to the Delta Plus XL mass spectrometer. Carbon and nitrogen isotope ratios are reported as δ^13^C and δ^15^N values in parts per thousand (per mil, ‰) relative to the VPDB and AIR standards, respectively (Table 2). Precision of analysis is generally ± 0.1‰ for δ^13^C and 0.2‰ for δ^15^N. These isotope values were compared to those of bone collagen of archaeological human and dog populations from the American Bottom from the Late Woodland, Terminal Late Woodland, Early Mississippian, and Late Mississippian Moorehead Phase periods (Supplemental Table 6)^74–77,108,109^. We calculated the diet %C_4_ using the following equation:

1 $$\% {\text{C}}_{4} = \left( {\left( { - 26.5 - \left( {^{13} {\text{C}}_{{{\text{coprolite}}}} - 0.9} \right)} \right)/15} \right)* - 100.$$

For this calculation, we used δ^13^C values of − 26.5‰ and − 11.5‰ for the pre-industrial averages of C_3_ and C_4_ plants to reflect diets of 0% C_4_ and 100% C_4_, respectively^110^, and used an offset between diet and feces of − 0.9^12^.

### Macroscopic analyses

Subsequent to DNA sample extraction, the remainder of each coprolite was submitted to the Archaeobotany Section of the Illinois State Archaeological Survey (ISAS), Prairie Research Institute, at the University of Illinois at Urbana-Champaign for macrobotanical analysis. The coprolites were all too solid (brick-like hardness) to permit easy disaggregation. Therefore, to facilitate the extraction of any existing macrobotanical remains, an attempt was made to soften and disperse matrix by placing them in a glass vial with distilled water. Up to 1 tsp of sodium phosphate tribasic dodecahydrate was added to the water in hopes that matrix substrate would deflocculate, and samples were allowed to soak for varying times (Supplemental Table 2). However, after six days of soaking, this method was unsuccessful in dispersing the matrix for any sample. Samples were then removed from the water and allowed to dry completely. Gentle pressure was used to break the samples up into small pieces. The resultant materials were then examined under low magnification (8× to 60×) using a stereoscopic binocular microscope. Given the magnification used, the lack of matrix dispersal likely did not impact the results. No plant materials were identified in the samples, however small bones and fish scales were observed. Samples were then transferred to the ISAS Zooarchaeology Section for further analysis.

## Supplementary Information

Supplementary Information

## Data Availability

The sequencing reads generated for this project are available at the Short Read Archive as BioProject PRJNA657304. The scripts and pipeline for read processing and diet taxonomy analyses are available at https://github.com/kelsey-witt/diet-taxonomy-pipeline, and the pipeline for the microbiome analyses are available at https://github.com/kyarlagadda/paleofecal_microbiome_pipeline.
