# Author Correction: Integrative analysis of DNA, macroscopic remains and stable isotopes of dog coprolites to reconstruct community diet

**DOI:** 10.1038/s41598-021-90889-x

**Published:** 2021-05-26

**Authors:** Kelsey E. Witt, Karthik Yarlagadda, Julie M. Allen, Alyssa C. Bader, Mary L. Simon, Steven R. Kuehn, Kelly S. Swanson, Tzu-Wen L. Cross, Kristin M. Hedman, Stanley H. Ambrose, Ripan S. Malhi

**Affiliations:** 1 Program in Ecology, Evolution and Conservation Biology, University of Illinois at Urbana-Champaign, Urbana-Champaign, IL USA; 2 Ecology and Evolutionary Biology and Center for Computational and Molecular Biology, Brown University, Providence, RI USA; 3 Department of Anthropology, University of Illinois at Urbana-Champaign, Urbana-Champaign, IL USA; 4 Biology Department, University of Nevada Reno, Reno, NV USA; 5 Sealaska Heritage Institute, Juneau, AK USA; 6 Illinois State Archaeological Survey, Prairie Research Institute, University of Illinois at Urbana-Champaign, Urbana, IL USA; 7 Department of Animal Sciences, University of Illinois at Urbana-Champaign, Urbana, IL USA; 8 Division of Nutritional Sciences, University of Illinois at Urbana-Champaign, Urbana, IL USA; 9 Department of Veterinary Clinical Medicine, University of Illinois at Urbana-Champaign, Urbana, IL USA; 10 Carl R. Woese Institute for Genomic Biology, University of Illinois at Urbana-Champaign, Urbana, IL USA

Correction to: *Scientific Reports* 10.1038/s41598-021-82362-6, published online 04 February 2021

The original version of this Article contained an error in the Discussion section where,

“Stable isotope results from the coprolites suggest a diet of terrestrial fauna, fish with low δ^15^N and high δ^13^C values, and C_3_ plants with the possible addition of C_4_ plants such as maize and/or maygrass.”

now reads:

“Stable isotope results from the coprolites suggest a diet of terrestrial fauna, fish with low δ^15^N and high δ^13^C values, and C_3_ plants with the possible addition of C_4_ plants such as maize.”

The original Article has been corrected.

