# Supplementary material for: Integrative analysis of DNA, macroscopic remains and stable isotopes of dog coprolites to reconstruct community diet
Source: Sci Rep. 2021 Feb 4;11:3113. doi: 10.1038/s41598-021-82362-6 (PMC7862676; doi:10.1038/s41598-021-82362-6)
Supplement: Supplementary file 1 — Supplementary Information [file 41598_2021_82362_MOESM1_ESM.pdf]

Integrative analysis of DNA, macroscopic remains and stable isotopes of dog coprolites to reconstruct community diet

Supplementary Materials

Kelsey E. Witt*[1-2], Karthik Yarlagadda[3], Julie M. Allen[4], Alyssa C. Bader[3,5], Mary L. Simon[6], Steven R. Kuehn[6], Kelly S. Swanson[7-9], Tzu-Wen L. Cross[8], Kristin M. Hedman[6], Stanley H. Ambrose[3], Ripan S. Malhi[1,3,10]

1: Program in Ecology, Evolution and Conservation Biology, University of Illinois at Urbana-Champaign, Urbana-Champaign, IL, USA

2: Ecology and Evolutionary Biology and Center for Computational and Molecular Biology, Brown University, Providence, RI, USA

3: Department of Anthropology, University of Illinois at Urbana-Champaign, Urbana-Champaign, IL, USA

4: Biology Department, University of Nevada Reno, Reno, NV, USA

5: Sealaska Heritage Institute, Juneau, AK, USA

6: Illinois State Archaeological Survey, Urbana, IL, USA

7: Department of Animal Sciences, University of Illinois at Urbana-Champaign, Urbana, IL, USA

8: Division of Nutritional Sciences, University of Illinois at Urbana-Champaign, Urbana, IL, USA

9: Department of Veterinary Clinical Medicine, University of Illinois at Urbana-Champaign, Urbana, IL, USA

10: Carl R. Woese Institute for Genomic Biology, University of Illinois at Urbana-Champaign, Urbana, IL, USA

*Corresponding author: Kelsey E. Witt, kelsey_witt_dillon@brown.edu

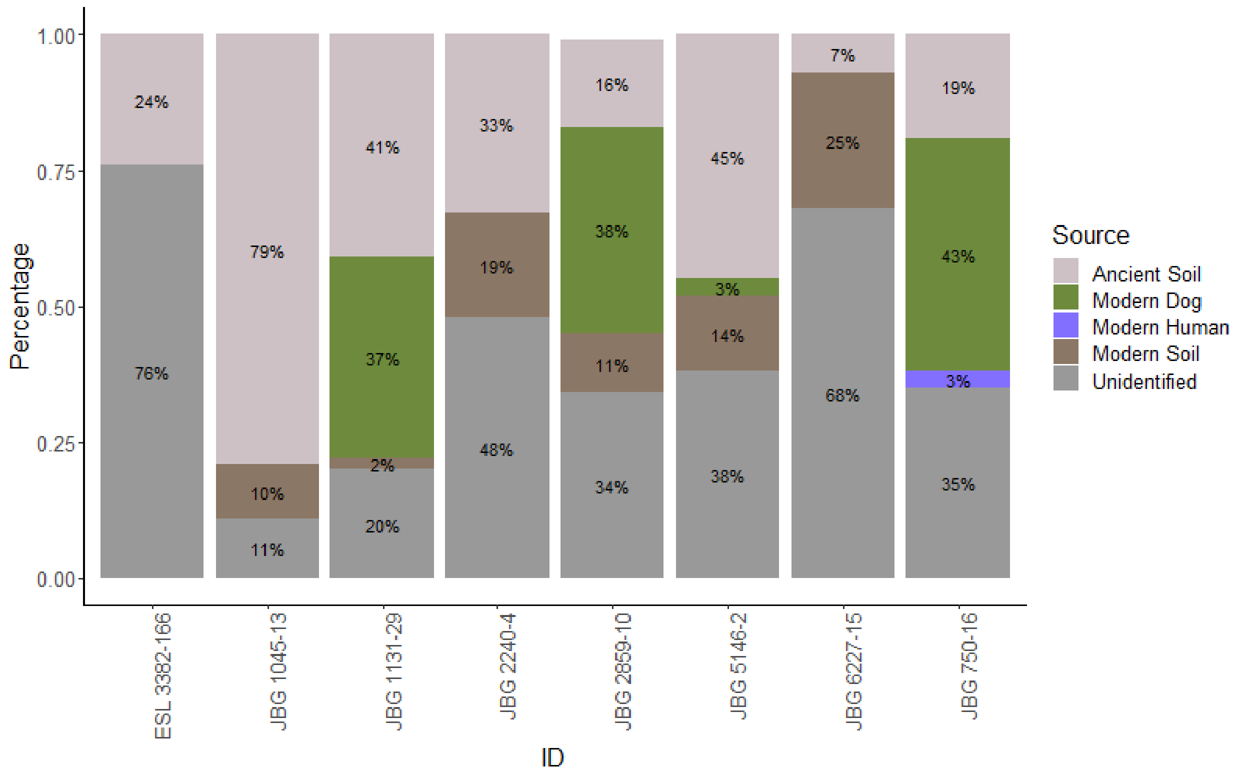

Supplemental Figure 1: SourceTracker results, indicating soil sources and matches to modern dog or human fecal microbiomes in the coprolites. The unidentified portions represent reads that could not be assigned to one of the listed sources (soil contaminants, modern dog microbiome, or modern human microbiome).

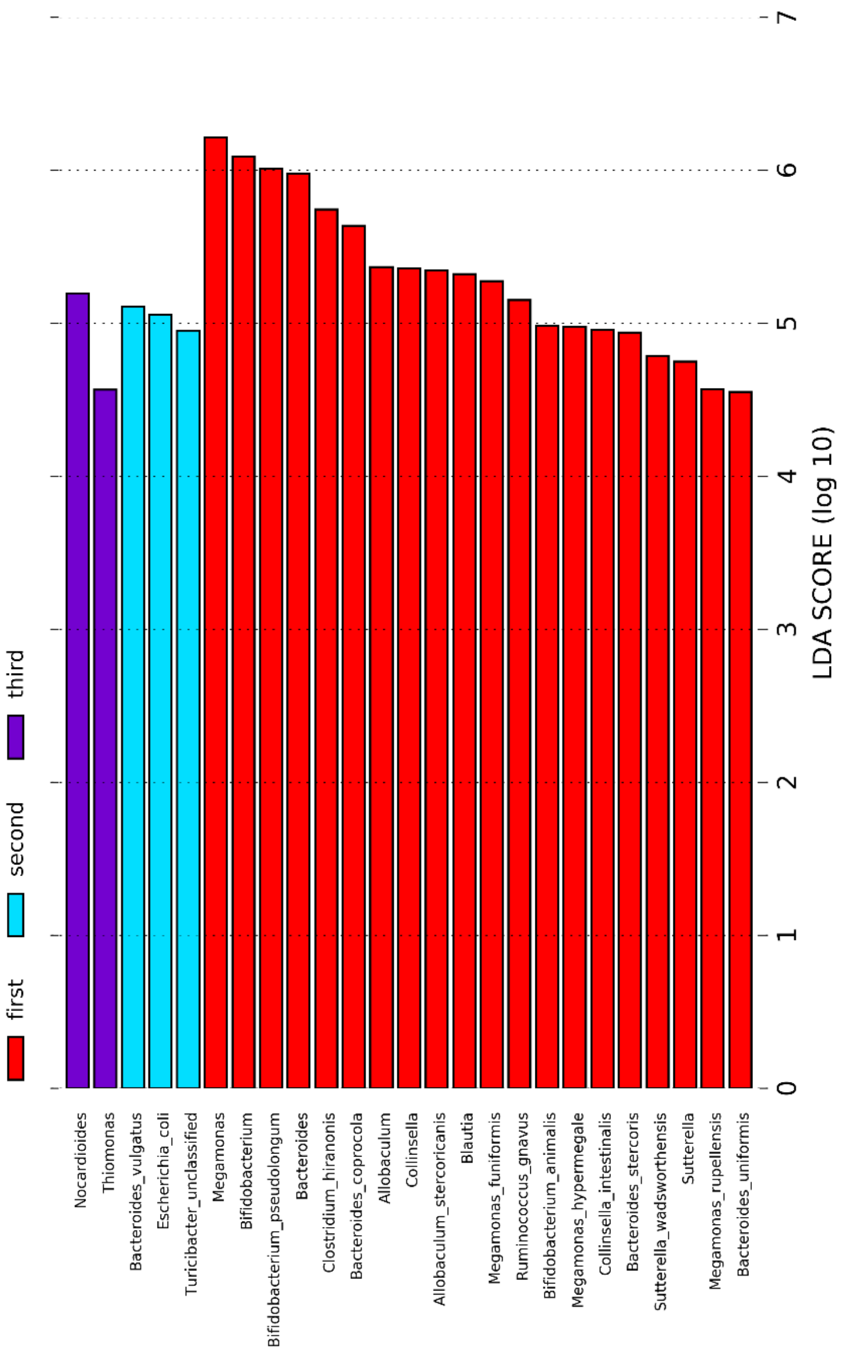

Supplemental Figure 2: LEfSe results when coprolit and fecal samples are grouped by the clusters shown in the PCA. Three clusters were defined by this method: “first” refers to the cluster of modern dogs on the low protein diet; “second” refers to the modern dogs on the high protein diet and the singular coprolites (JBG 750-16); “third refers to the remaining coprolites.

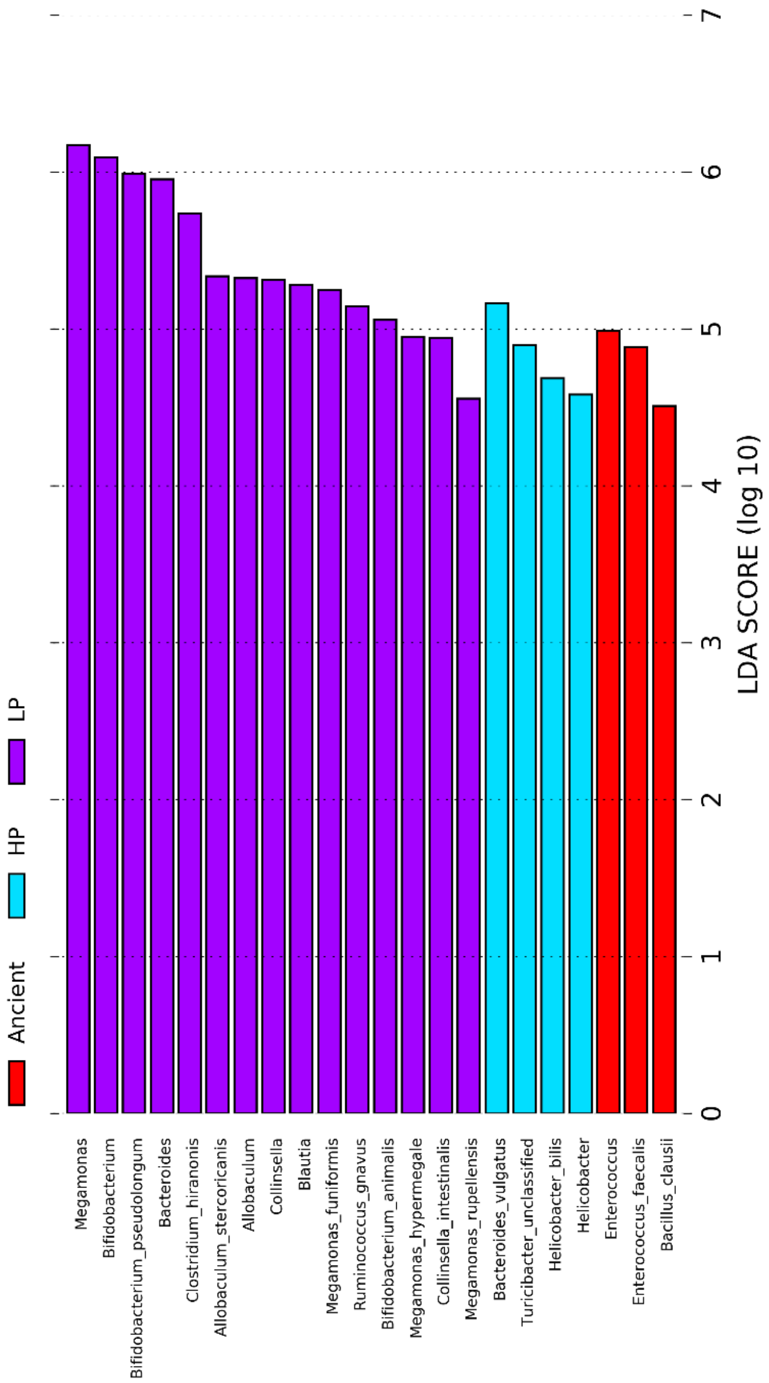

Supplemental Figure 3: LEfSe results when coprolite and fecal samples are grouped by known clusters, grouping all of the coprolites together, and separating the modern dogs by diet. HP and LP refer to the high and low protein diets respectively.

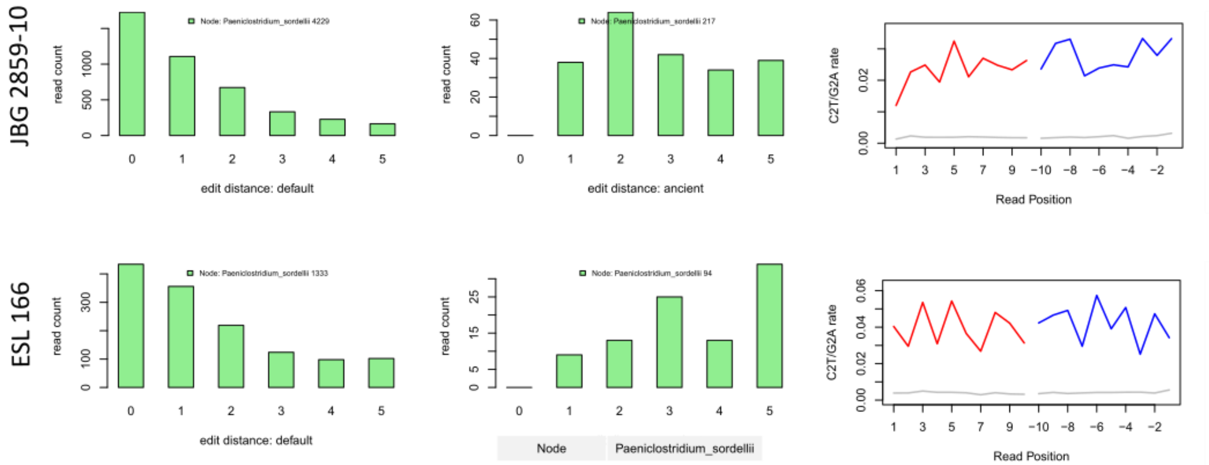

Supplemental Figure 4: A summary of the HOPS results for *C. sordellii* in the coprolites with an ancient signature for the pathogen. Each row is a different sample, and the plots depict, in order, the number of reads mapping per node, the number of ancient reads mapping per node, and the damage pattern of the ancient reads.

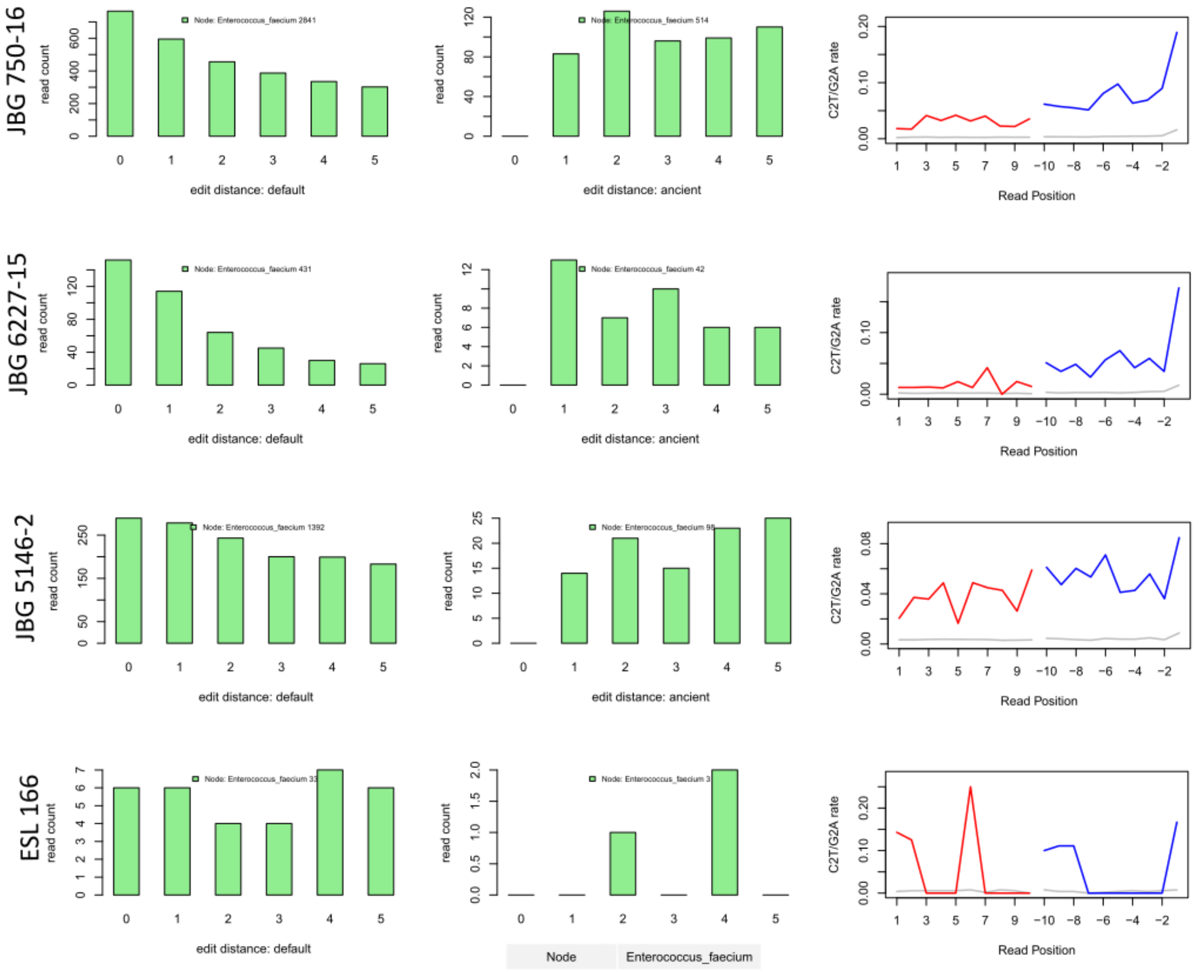

Supplemental Figure 5: A summary of the HOPS results for *E. faecium* in the coprolites with an ancient signature for the pathogen. Each row is a different sample, and the plots depict, in order, the number of reads mapping per node, the number of ancient reads mapping per node, and the damage pattern of the ancient reads.

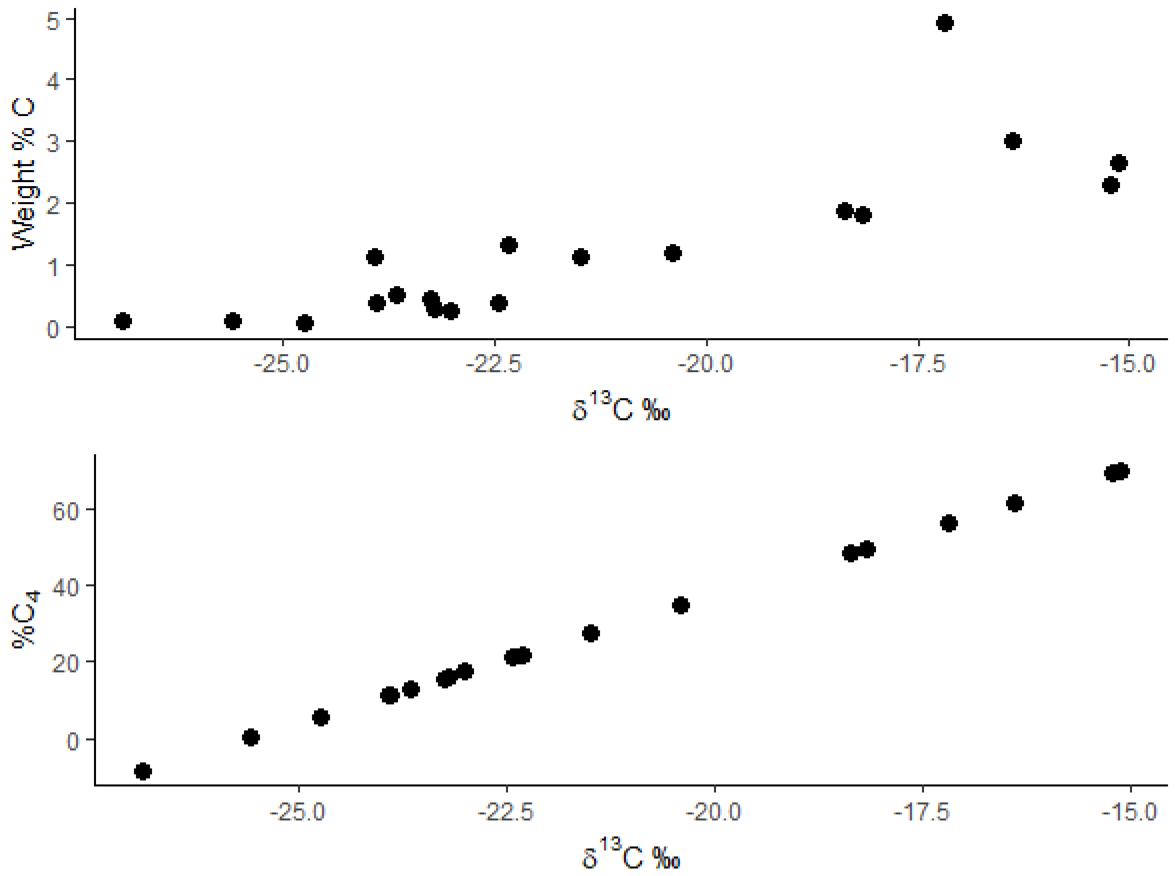

Supplemental Figure 5: The association between $\delta^{13}C$ and weight percent C and % C_4, respectively, for the coprolites.

Supplementary Tables

Supplemental Table 1: A list of plant and animal taxa recovered from American Bottom archaeological sites during the Late Woodland and Terminal Late Woodland periods.

Type	Scientific Name	Common Name
**Plants**		
Fruits	*Asimina triloba*	Pawpaw
	Morus rubra	Mulberry
	Prunus spp.	Plum/Cherry
	Rhus spp.	Sumac
	Vitis riparia	Wild grape
Nuts	*Carya spp.*	Hickory
	Corylus americana	Hazelnut
	Juglans nigra	Walnut
	Quercus spp.	Acorn
Domesticated/Cultivated Plants	*Chenopodium berlandieri*	Goosefoot
	Cucurbita pepo	Squash
	Helianthus annuus	Sunflower
	Hordeum pusillum	Little Barley
	Iva annua	Sumpweed
	Nicotiana spp.	Tobacco
	Phalaris caroliniana	Maygrass
	Polygonum erectum	Erect Knotweed
	Zea mays	Maize
Wild Plants	*Amaranthus spp.*	Amaranth
	Desmodium spp.	Tick Clover
	Eleusine indica	Goosegrass
	Ipomoea spp.	Wild Morning Glory
	Panicum spp.	Panic Grass
	Strophostyles helvola	Wild Bean
	Portulaca spp.	Purslane
	Solanum spp.	Nightshade
**Animals**		
Mammals	*Castor canadensis*	Beaver
	Cervus elephas	Elk
	Geomys bursarius	Pocket gopher
	Odocoileus virginianus	White-Tailed Deer
	Ondatra zibethicus	Muskrat
	Procyon lotor	Raccoon
	Sciurus spp.	Squirrel
	Sylvilagus floridanus	Rabbit
Birds	*Aix sponsa*	Wood Duck
	Anas acuta	Pintail
	Anas americana	Widgeon
	Anas platyrhynchos	Mallard
	Anas crecca/discors	Teal
	Aythya americana	Redhead

	Aythya collaris	Ring-Necked Duck
	Branta canadensis	Canada Goose
	Bucephala spp.	Goldeneye/Bufflehead
	Chen caerulescens	Snow Goose
	Family Podicipedidae	Grebe
	Family Rallidae	Rail
	Fulica americana	American Coot
	Grus canadensis	Sandhill Crane
	Meleagris gallopavo	Turkey
	Lophodytes spp.	Merganser
	Mergus spp.	Merganser
	Oxyura jamaicensis	Ruddy Duck
	Tringa spp.	Yellowlegs
	Tympanuchus cupido	Prairie Chicken
	T. phasianellus	Sharp-Tailed Grouse
Fish	*Dorosoma cepedianum*	Gizzard Shad
	Ictalurus punctatus	Channel Catfish
	Ameiurus spp.	Bullhead
	Family Ictaluridae	Catfish
	Ictiobus cyprinellus	Bigmouth Buffalo
	Perca flavescens	Yellow Perch
	Sander vitreus	Walleye
	Family Cyprinidae	Minnow
	Family Catostomidae	Suckers
	Lepomis spp.	Sunfish
	Micropterus spp.	Bass
	Esox spp.	Pike
	Aplodinotus grunniens	Freshwater Drum
	Atractosteus spatula	Gar
	Amia calva	Bowfin
	Acipenser fulvescens	Sturgeon
Shellfish	Class Bivalvia*	Freshwater Mussels

Supplemental Table 2: A summary of the macroscopic results, detailing the processing and botanical/faunal remains identified

COPROLITE	ESTIMATED WEIGHT (G)	SOAK DURATION (DAYS)	SUCCESS LEVEL	POST- SOAK COMMENT	BOTANICAL REMAINS IDENTIFIED	FAUNAL REMAINS IDENTIFIED
**JBG 750-16**	16.1	1	Moderate	Some breakage	No	11: indet. bird, fish, and vertebrate
**JBG 1045-13**	14.5	1	No	Large, hard mass	No	113: *Lepisosteus* and indet. fish and vertebrates
**JBG 1131-29**	4.8	7	Moderate	Some breakage	No	11: indet. fish and vertebrate
**JBG 2240-4**	18	7	Moderate	Some breakage	No	No
**JBG 2859-10**	4.5	1	Moderate	Some breakage	No	8: *Ameiurus* and indet fish and vertebrate
**JBG 5146-2**	2.5	7	No	Large, hard mass	No	41: indet. fish and vertebrates
**JBG 6553-2**	9.2	1	Yes	Broken down to soil	No	No
**JBG 6227-15**	7.9	7	No	Large, hard mass	No	1: indet. vertebrate
**JBG 7186-2**	4	7	No	Large, hard mass	No	9: indet. vertebrate
**JBG 7298-2**	2.4	7	Moderate	Some breakage	No	38: *Ameiurus* and indet. fish and vertebrate
**ESL 3382-166**	4.4	1	No	Large, hard mass	No	No

Supplemental Table 3: A summary of the DNA sequencing results, including counts of raw, trimmed, and assembled reads, as well as dietary and microbial hits.

SAMPLE	TYPE	ANALYZED FOR DNA?	RAW READS	TRIMMED READS	ASSEMBLED READS	DIETARY READS	MICROBIAL READS
**JBG 750-16**	Coprolite	Yes	79,691,878	29,469,223	3,164,239	306	1,656,085
**JBG 1045-13**	Coprolite	Yes	83,314,584	26,206,075	3,110,977	19	307,943
**JBG 1131-29**	Coprolite	Yes	66,847,891	33,569,605	1,923,729	7	528,303
**JBG 2240-4**	Coprolite	Yes	62,474,161	10,557,502	1,525,823	21	14,814
**JBG 2859-10**	Coprolite	Yes	64,729,787	31,303,216	1,944,621	137	267,787
**JBG 5146-2**	Coprolite	Yes	102,139,995	42,358,981	2,752,170	39	146,584
**JBG 6227-15**	Coprolite	Yes	72,375,269	40,565,183	3,325,616	17	542,872
**JBG 6553-2**	Coprolite	No	-	-	-	N/A	N/A
**JBG 7186-2**	Coprolite	No	-	-	-	N/A	N/A
**JBG 7298-2**	Coprolite	Yes	44,305,225	1,594,550	130,340	2	0
**ESL166**	Coprolite	Yes	40,609,814	7,705,867	583,487	4	232,363
**JBG2**	Bone Control	Yes	8,131,302	7,525,498	196,496	0	N/A
**JBG47**	Bone Control	Yes	57,091,326	2,476,144	251,131	0	2,148
**F3**	Soil Control	Yes	25,834,112	25,017,361	N/A	N/A	27,034
**F7**	Soil Control	Yes	13,682,743	13,233,423	N/A	N/A	8,318
**AM2D**	Fecal	Yes	45,256,484	43,439,627	N/A	N/A	8,855,513
**AM4C**	Fecal	Yes	33,882,273	32,568,262	N/A	N/A	3,052,759
**BR3C**	Fecal	Yes	33,681,305	32,722,945	N/A	N/A	4,187,362
**BR4D**	Fecal	Yes	48,747,732	46,628,002	N/A	N/A	19,715,356
**HO1C**	Fecal	Yes	39,049,867	37,934,312	N/A	N/A	968,147
**HO3D**	Fecal	Yes	54,867,155	52,713,008	N/A	N/A	17,095,148
**SH1D**	Fecal	Yes	47,838,183	46,098,042	N/A	N/A	13,735,554
**SH2C**	Fecal	Yes	39,210,343	36,881,042	N/A	N/A	1,549,805

Supplementary Table 4: The read counts of the taxa recovered from DNA sequencing of the coprolites, per genus and per sample. * indicates that the sample is a negative control.

SAMPLE	ESL 166	JBG C4	JBG C10	JBG C13	JBG C15	JBG C16	JBG C29	JBG C82	JBGC 462	JBG2*	JBG47*
**Lepisosteus**	0	0	0	13	0	0	0	0	0	0	0
**Lepomis**	0	0	0	0	0	0	0	0	6	0	0
**Micropterus**	0	0	0	0	0	0	0	0	2	0	0
**Perca**	0	0	0	0	0	0	0	0	2	0	0
**Ictalurus**	0	0	0	0	0	0	1	0	1	0	0
**Cyprinus**	0	0	0	0	2	0	1	0	1	0	0
**Homo**	99	84	61	53	26	117	1	7	23	151	18
**Canis**	3	0	61	50	8	101	62	10	1206	5	2
**Anas**	0	0	0	0	0	0	0	0	2	0	0
**Anser**	0	0	0	0	0	0	0	0	6	0	0
**Rana**	1	0	0	0	2	0	4	2	5	0	0
**Toxocara**	0	0	129	0	0	296	0	0	0	0	0
**Pristionchus**	0	0	0	0	0	0	0	0	11	0	0
**Thelazia**	0	0	0	0	0	0	0	0	2	0	0
**Xiphinema**	0	0	2	0	0	0	0	0	0	0	0
**Dendroctonus**	3	0	0	0	0	0	0	0	0	0	0
**Heteromita**	0	1	0	0	3	0	0	0	0	0	0
**Ipomoea**	0	1	0	1	0	0	0	0	0	0	0
**Nicotiana**	0	2	0	0	1	0	0	0	0	0	0
**Hyoscyamus**	0	0	0	1	0	0	0	0	0	0	0
**Solanum**	6	66	0	58	0	71	12	5	15	0	13
**Glycine**	0	6	1	2	0	0	0	0	0	0	0
**Juglans**	0	1	1	0	0	0	0	0	1	0	0
**Vitis**	0	0	4	0	4	10	0	0	0	0	0
**Triticum**	0	10	0	2	5	0	1	0	0	0	0

Supplementary Table 5: A summary of the HOPS results for each putatively pathogenic species in each coprolite. The percentages indicate a % match between a read and the species' genome. Bold names indicate species with a signature consistent with ancient DNA.

SAMPLE	BACTERIAL SPECIES	80%	85%	90%	95%	100%	TOTAL
**JBG 750-16**	Bacillus_clausii	0	0	0	0	0	0
	Bacillus_clausii_KSM-K16	0	0	25	130	49	204
	Clostridium_colicanis	0	0	0	0	0	0
	Clostridium_colicanis_DSM_13634	0	0	174	142	1	317
	Enterococcus_casseliflavus	0	0	0	0	0	0
	Enterococcus_casseliflavus_EC20	0	0	129	488	97	714
	Enterococcus_faecalis	0	0	0	0	0	0
	Enterococcus_faecalis_EnGen0107	0	0	55	447	145	647
	**Enterococcus_faecium**	**0**	**0**	**220**	**462**	**83**	**765**
	Enterococcus_hirae	0	0	0	0	0	0
	Enterococcus_hirae_ATCC_9790	0	0	25	380	238	643
	**Paeniclostridium_sordellii**	**0**	**0**	**401**	**277**	**21**	**699**
	Total			1029	2326	634	
**JBG 1045-13**	Bacillus_clausii	0	0	0	0	0	0
	Bacillus_clausii_KSM-K16	0	0	7	10	4	21
	Clostridium_colicanis	0	0	0	0	0	0
	Clostridium_colicanis_DSM_13634	0	0	13	2	0	15
	Enterococcus_casseliflavus	0	0	0	0	0	0
	Enterococcus_casseliflavus_EC20	0	0	5	18	6	29
	Enterococcus_faecalis	0	0	0	0	0	0
	Enterococcus_faecalis_EnGen0107	0	0	13	150	48	211
	**Enterococcus_faecium**	**0**	**0**	**8**	**11**	**2**	**21**
	Enterococcus_hirae	0	0	0	0	0	0
	Enterococcus_hirae_ATCC_9790	0	0	1	42	28	71
	**Paeniclostridium_sordellii**	**0**	**0**	**346**	**240**	**18**	**604**
	Total			393	473	106	
**JBG 1131-29**	Bacillus_clausii	0	0	0	0	0	0
	Bacillus_clausii_KSM-K16	0	0	13	39	16	68
	Clostridium_colicanis	0	0	0	0	0	0
	Clostridium_colicanis_DSM_13634	0	0	39	17	0	56
	Enterococcus_casseliflavus	0	0	0	0	0	0
	Enterococcus_casseliflavus_EC20	0	0	65	204	31	300
	Enterococcus_faecalis	0	0	0	0	0	0
	Enterococcus_faecalis_EnGen0107	0	0	13	262	116	391
	**Enterococcus_faecium**	**0**	**0**	**79**	**122**	**12**	**213**
	Enterococcus_hirae	0	0	0	0	0	0
	Enterococcus_hirae_ATCC_9790	0	0	26	154	72	252
	**Paeniclostridium_sordellii**	**0**	**0**	**364**	**233**	**22**	**619**
	Total			599	1031	269	

**JBG 2240-4**	Bacillus_clausii	0	0	0	0	0	0
	Bacillus_clausii_KSM-K16	0	0	27	165	39	231
	Clostridium_colicanis	0	0	0	0	0	0
	Clostridium_colicanis_DSM_13634	0	0	5	3	0	8
	Enterococcus_casseliflavus	0	0	0	0	0	0
	Enterococcus_casseliflavus_EC20	0	0	0	1	0	1
	Enterococcus_faecalis	0	0	0	0	0	0
	Enterococcus_faecalis_EnGen0107	0	0	4	36	17	57
	**Enterococcus_faecium**	**0**	**0**	**1**	**4**	**0**	**5**
	Enterococcus_hirae	0	0	0	0	0	0
	Enterococcus_hirae_ATCC_9790	0	0	3	17	7	27
	**Paeniclostridium_sordellii**	**0**	**0**	**6**	**6**	**0**	**12**
	Total			46	232	63	
**JBG 2859-10**	Bacillus_clausii	0	0	0	0	0	0
	Bacillus_clausii_KSM-K16	0	0	24	71	27	122
	Clostridium_colicanis	0	0	0	0	0	0
	Clostridium_colicanis_DSM_13634	0	0	37	14	0	51
	Enterococcus_casseliflavus	0	0	0	0	0	0
	Enterococcus_casseliflavus_EC20	0	0	111	381	52	544
	Enterococcus_faecalis	0	0	0	0	0	0
	Enterococcus_faecalis_EnGen0107	0	0	23	289	122	434
	**Enterococcus_faecium**	**0**	**0**	**311**	**462**	**33**	**806**
	Enterococcus_hirae	0	0	0	0	0	0
	Enterococcus_hirae_ATCC_9790	0	0	11	116	55	182
	**Paeniclostridium_sordellii**	**0**	**0**	**108**	**175**	**38**	**321**
	Total			625	1508	327	
**JBG 5146-2**	Bacillus_clausii	0	0	0	0	0	0
	Bacillus_clausii_KSM-K16	0	0	9	15	10	34
	Clostridium_colicanis	0	0	0	0	0	0
	Clostridium_colicanis_DSM_13634	0	0	7	2	0	9
	Enterococcus_casseliflavus	0	0	0	0	0	0
	Enterococcus_casseliflavus_EC20	0	0	14	23	6	43
	Enterococcus_faecalis	0	0	0	0	0	0
	Enterococcus_faecalis_EnGen0107	0	0	4	53	16	73
	**Enterococcus_faecium**	**0**	**0**	**72**	**107**	**14**	**193**
	Enterococcus_hirae	0	0	0	0	0	0
	Enterococcus_hirae_ATCC_9790	0	0	12	55	37	104
	**Paeniclostridium_sordellii**	**0**	**0**	**303**	**191**	**13**	**507**
	Total			421	446	96	
**JBG 6227-15**	Bacillus_clausii	0	0	0	0	0	0
	Bacillus_clausii_KSM-K16	0	0	12	21	10	43
	Clostridium_colicanis	0	0	0	0	0	0
	Clostridium_colicanis_DSM_13634	0	0	40	29	0	69
	Enterococcus_casseliflavus	0	0	0	0	0	0

	Enterococcus_casseliflavus_EC20	0	0	4	6	1	11
	Enterococcus_faecalis	0	0	0	0	0	0
	Enterococcus_faecalis_EnGen0107	0	0	15	93	40	148
	**Enterococcus_faecium**	**0**	**0**	**9**	**30**	**13**	**52**
	Enterococcus_hirae	0	0	0	0	0	0
	Enterococcus_hirae_ATCC_9790	0	0	22	257	179	458
	**Paeniclostridium_sordellii**	**0**	**0**	**327**	**197**	**15**	**539**
	Total			429	633	258	
**JBG 7298-2**	Bacillus_clausii	0	0	0	0	0	0
	Bacillus_clausii_KSM-K16	0	0	0	1	0	1
	Clostridium_colicanis	0	0	0	0	0	0
	Clostridium_colicanis_DSM_13634	0	0	0	0	0	0
	Enterococcus_casseliflavus	0	0	0	0	0	0
	Enterococcus_casseliflavus_EC20	0	0	0	0	0	0
	Enterococcus_faecalis	0	0	0	0	0	0
	Enterococcus_faecalis_EnGen0107	0	0	0	0	0	0
	**Enterococcus_faecium**	**0**	**0**	**0**	**0**	**0**	**0**
	Enterococcus_hirae	0	0	0	0	0	0
	Enterococcus_hirae_ATCC_9790	0	0	0	0	0	0
	**Paeniclostridium_sordellii**	**0**	**0**	**3**	**3**	**0**	**6**
	Total			3	4	0	
**ESL 166**	Bacillus_clausii	0	0	0	0	0	0
	Bacillus_clausii_KSM-K16	0	0	4	2	0	6
	Clostridium_colicanis	0	0	0	0	0	0
	Clostridium_colicanis_DSM_13634	0	0	7	1	0	8
	Enterococcus_casseliflavus	0	0	0	0	0	0
	Enterococcus_casseliflavus_EC20	0	0	1	3	1	5
	Enterococcus_faecalis	0	0	0	0	0	0
	Enterococcus_faecalis_EnGen0107	0	0	2	5	1	8
	**Enterococcus_faecium**	**0**	**0**	**0**	**4**	**0**	**4**
	Enterococcus_hirae	0	0	0	0	0	0
	Enterococcus_hirae_ATCC_9790	0	0	1	0	1	2
	**Paeniclostridium_sordellii**	**0**	**0**	**207**	**106**	**9**	**322**
	Total			222	121	12	

Supplemental Table 6: Human and dog bone collagen average $\delta^{15}N$ and $\delta^{13}C$ from American Bottom archaeological sites. LW: Late Woodland; TLW: Terminal Late Woodland; *: includes one dog from the American Bottom Cunningham Site. Diet % C_4 is calculated assuming $\delta^{13}C$ end member values of -26.5‰ for 0% C_4 and -11.5‰ for 100% C_4, and a diet-collagen difference of +5.0‰.

SITE	APPROX. DATE AD	COMPONENT (DATE AD)	AVERAGE $\delta 13C_{COLLAGEN}$ (‰)	± 1 SD	AVERAGE $\delta 15N_{COLLAGEN}$ (‰)	± 1 SD	SAMPLE SIZE	DIET %C4	SOURCE
**HUMANS**									
KANE MOUNDS	600-800	Late Woodland	-20.6	0.29	8.6	0.77	6	6	(54)
SCHILD	600-900	Late Woodland	-20.5	0.21	9.25	1.01	4	6.7	(54)
JANEY B. GOODE	700-900	LW/TLW (~800)	-20.1	-	9	-	1	9.3	(54)
DRDA	800-1000	TLW (~900)	-17.4	0.28	8.7	0	2	27.3	(109)
LILLIE	800-1000	TLW (~900)	-20.5	0.2	9.8	0.71	3	6.7	(77)
CAHOKIA TRACT 15B	800-1000	TLW/E Miss (~900)	-20.4	0.11	8	0.1	3	7.3	(75)
ESTL	800-1000	TLW (~900)	-19.9	0	9	0	1	10.7	(76)
JANEY B. GOODE	1000-1200	Early Mississippian	-11.2	0.96	9	0.2	3	68.7	(54)
ESTL	1000-1200	Early Mississippian	-13.7	1.68	9	0.26	46	52	(76)
MOUND 72	1000-1200	Early Mississippian	-16.6	2.1	9	1.34	9	32.7	(108)
JANEY B. GOODE	1200-1350	Moorehead/Sand Prairie	-11.3	0.73	8.5	0.29	4	68	(54)
ESTL	1200-1300	Moorehead/Sand Prairie	-12.3	1.77	9	0	6	61.3	(76)
CAHOKIA TRACK 15B	1200-1300	Moorehead/Sand Prairie	-11.3	1.13	9	1.06	2	68	(75)
EAST ST LOUIS QUARRY	1200-1300	Moorehead (~1275)	-11	1.1	9.1	0.64	21	70	(74)
FLORENCE STREET	1200-1300	Moorehead (~1275)	-11.2	0.96	9.9	0.44	9	68.7	(74)
RANGE	1200-1300	Moorehead (~1275)	-11.4	1.5	9.4	0.83	6	67.3	(74)
CORBIN MOUNDS	1200-1300	Moorehead (~AD 1275)	-12.1	1.58	9.1	0.35	13	62.7	(74)
**DOGS**									
JANEY B. GOODE*	600-800	LW	-20.6	0.5	8.8	0.8	4	6	(54)
JANEY B. GOODE	800-1000	TLW	-16.1	3.6	8.8	8.08	5	36	(54)
JANEY B. GOODE	1000-1200	Early Mississippian	-15.1	3.1	8.8	0.5	6	42.7	(54)
